# Recent Advances and Challenges of Textile-Based Triboelectric Nanogenerators for Smart Healthcare and Sports Applications

**DOI:** 10.3390/nano16020141

**Published:** 2026-01-21

**Authors:** Lijun Chen, Jie Wu, Ke Xu, Yuanyuan Zhang, Chaoyu Chen

**Affiliations:** 1 Digital Technology and Innovation Design, Jiangnan University, Wuxi 214122, China; lijunchen@jiangnan.edu.cn (L.C.); 1094220226@stu.jiangnan.edu.cn (J.W.); 1094230216@stu.jiangnan.edu.cn (K.X.); 1090724306@stu.jiangnan.edu.cn (Y.Z.); 2 Engineering Research Center of Knitting Technology, Ministry of Education, College of Textile Science and Engineering, Jiangnan University, Wuxi 214122, China

**Keywords:** textile-based triboelectric nanogenerators, smart healthcare, pulse monitoring, sleep monitoring, respiratory monitoring, motion monitoring

## Abstract

The combination of nanogenerator technology and traditional textile materials has given rise to textile-based triboelectric nanogenerators (T-TENGs) structured from fibers, yarns, and fabrics. Due to their lightweight, flexibility, washability, and cost-effectiveness, T-TENGs offer a promising platform for powering and sensing in next-generation wearable electronics, with particularly significant potential in smart healthcare and sports monitoring. However, the inherent electrical and structural limitations of textile materials often restrict their power output, signal stability, and sensing range, making it challenging to achieve both high electrical performance and high sensing sensitivity. This review focuses on the application of T-TENGs in smart healthcare and sports. It systematically presents recent developments in textile material selection, sensing structure, fabric design, working mechanisms, accuracy optimization, and practical application scenarios. Furthermore, it provides a critical analysis of the recurring structural and material limitations that constrain performance and offers constructive pathways to address them. Key challenges such as the low charge density of textile interfaces may be mitigated by selecting low-hygroscopicity materials, applying hydrophobic treatments, and optimizing textile structures to enhance contact efficiency and environmental stability. Issues of signal instability under dynamic deformation call for advanced structural designs that accommodate strain without compromising electrical pathways, coupled with robust signal processing algorithms. By providing a comparative analysis across materials and structures, this review aims to inform future designs and accelerate the translation of high-performance T-TENGs from laboratory research to real-world implementation.

## 1. Introduction

Wearable electronic devices are increasingly integrated with the Internet of Things, big data, and artificial intelligence, accelerating their transformation toward intelligent integration [1,2,3,4]. In the field of smart healthcare, they support continuous monitoring of vital signs, early disease warnings, and personalized rehabilitation treatments [5,6,7,8]. In sports and fitness, they facilitate detailed exercise analysis, real-time training guidance, and injury prevention [9,10,11]. However, these devices face significant challenges due to the limitations of traditional lithium-ion battery technology, such as insufficient energy density, safety concerns, and limited battery life, which hinder long-term continuous monitoring and wearability [12,13]. The industry is actively seeking breakthroughs through material innovations (e.g., solid-state batteries, micro-nuclear batteries), process advancements (e.g., laminated and curved battery technologies), and energy harvesting (e.g., self-powered technologies) to promote lighter, longer-lasting, and safer devices [14,15,16,17]. Future developments will further expand applications in healthcare monitoring and sports behavior optimization, while enhancing personalized customization and cross-platform interoperability to address challenges such as data privacy and device comfort, thereby unlocking their vast potential in smart healthcare, sports, and related fields [17,18,19,20].

To overcome this energy supply challenge, TENG technology has emerged with unique advantages [8,21,22,23]. Based on the coupling of triboelectrification and electrostatic induction, TENG efficiently harvests low-frequency, low-amplitude mechanical energy from the environment, such as human motion and respiratory vibrations [24,25,26,27,28]. Its peak power density has reached to 50 mW/cm^2^, enabling continuous self-powering through low-frequency mechanical energy like friction, thereby significantly enhancing its applicability [24,29,30,31]. Compared to traditional electromagnetic power generation, TENG demonstrates superior efficiency in low-frequency energy conversion, broader material selection options, and greater structural design flexibility [22,23,32,33,34]. Particularly noteworthy is its integration with textile materials. Through well-designed textile structures, energy harvesting units can be seamlessly incorporated into fibers, yarns, and fabrics, forming what can be described as a “smart textile system” [35,36,37]. This integration not only preserves the inherent flexibility, breathability, and comfort of textiles but also enables seamless energy harvesting and sensing functionalities, offering a novel technological pathway for building all-day, unobtrusive self-powered wearable platforms [38,39,40,41,42,43].

Currently, T-TENGs have made significant strides in medical and sports applications [44,45,46]. In smart healthcare, researchers have developed various biocompatible T-TENG devices for continuous monitoring of key physiological parameters such as electrocardiographic signals, breathing patterns, and limb movements, with some studies progressing to clinical trials [41,47,48,49]. In sports science, smart sportswear and wearable devices can collect multi-dimensional data, including gait characteristics, exercise intensity, and joint angles, providing precise support for scientific training and athletic rehabilitation [50,51,52,53,54]. However, transitioning from laboratory prototypes to practical applications faces several challenges: textile materials have limited charge retention capabilities, and further improvements are needed in interface charge density and stability; the structural integrity and functional reliability of devices under long-term wear, frequent washing, and complex deformations remain inadequate; signal stability and noise resistance in multi-environmental interferences require enhancement; and issues such as material standardization, process consistency, and cost control in large-scale production urgently need resolution [17,36,43,53,55].

Against this backdrop, this paper systematically reviews the latest research progress of T-TENGs in smart healthcare and sports applications. It delves into material innovation, structural optimization, and practical application scenarios, with a focus on addressing current technological bottlenecks and barriers to industrial transformation. The aim is to provide a comprehensive reference for future research in this field, promoting the transition of T-TENG technology from proof-of-concept to practical applications and accelerating the industrialization of self-powered smart wearable systems.

To ensure a focused, representative, and state-of-the-art analysis, this review adheres to a defined set of literature selection criteria. Our survey prioritizes peer-reviewed research (primarily from 2018 onward) published in leading journals spanning materials science, nanotechnology, and flexible electronics (e.g., Advanced Materials, ACS Nano, Nano Energy, Advanced Functional Materials). The core inclusion criterion is the implementation of the triboelectric effect within a genuine textile platform—encompassing fibers, yarns, and fabrics—specifically for applications in smart healthcare (physiological monitoring) and sports science (motion analysis). We emphasize works that introduce significant innovations in textile-compatible material design, structural engineering, device integration, or system-level functionality. Studies providing substantial quantitative performance data are particularly highlighted to enable meaningful cross-comparison, as reflected in the comparative tables presented in later sections.

## 2. T-TENG Structures

Moving beyond application-centric descriptions, a fundamental understanding of T-TENGs necessitates an architectural perspective that traces the hierarchical integration of functional materials into wearable systems. This section systematically deconstructs T-TENGs into their foundational elements—fibers, yarns, and fabrics—examining the commonly used materials, fabrication strategies, and working principles at each level. As shown in Table 1, a materials-property-structure-performance relationship framework is adopted to elucidate how design choices at the micro-scale (e.g., material triboelectric series, surface morphology) dictate the macro-scale functionality (e.g., output, sensitivity, comfort) of the final textile device.

As functional building blocks, fibrous elements integrate triboactive materials with conductive components through electrospinning, wet-spinning, or coating techniques, thereby establishing the microstructural foundation of T-TENGs [56,57,58]. At the most fundamental level, functional fibers serve as the building blocks for T-TENGs. These are typically engineered by integrating tribo-active and conductive components into a single fiber structure. Common materials include synthetic polymers like polyimide (PI), polytetrafluoroethylene (PTFE), and polyvinylidene fluoride (PVDF) as negative tribo-layers, and nylon, silk, or polyamide (PA) as positive counterparts [59]. Conductive elements are introduced via coatings (e.g., silver nanowires, carbon nanotubes), doped composites (e.g., graphite/PDMS), or conductive cores (e.g., stainless-steel filament) [60,61]. Fabrication methods such as electrospinning, wet-spinning, and dip-coating are pivotal for creating fibers with tailored surface morphologies (e.g., porous, core-sheath, micro-needled) that enhance the contact area and charge density [35,62]. The working principle at this stage is the intrinsic triboelectrification between the fiber material and its counter surface, with performance heavily influenced by the material’s electron affinity, dielectric constant, and the engineered micro/nano-topography.

Yarns spun from functional fibers achieve robust mechanical integration of triboelectric layers and electrodes via twisting, wrapping, or braiding, thereby enabling one-dimensional structural extension and serving as a critical bridge linking discrete fibers to macroscopic fabrics. The assembly of functional fibers into yarns represents the first step towards textile processability and structural integrity. Y-TENGs integrate triboelectric and conductive elements into a continuous, flexible, and durable one-dimensional structure [63,64,65]. This is achieved through processes like twisting, wrapping, or braiding. For instance, a conductive core yarn (e.g., silver-coated polyamide) can be helically wrapped with a triboelectric filament (e.g., PTFE) to form a core–shell triboelectric yarn [66]. Alternatively, multiple functional fibers can be co-twisted. This architectural advance provides mechanical robustness, enables effective strain distribution, and allows for length-scalable production. The working mechanism evolves to involve contact-separation or lateral sliding between different material components within the yarn structure itself or between the yarn and an external object, generating electrical signals in response to tensile or bending deformations [67,68].

Ultimately, functional yarns or fibers are integrated into two- or three-dimensional fabrics through weaving, knitting, or nonwoven techniques, enabling the large-area, breathable, and wearable system integration of T-TENGs, whose performance is macroscopically governed by fabric structural design [37,69,70,71]. The integration of functional yarns or fibers into fabrics marks the culmination of T-TENG development, transforming discrete elements into practical, wearable systems [72,73,74]. Fabric-based T-TENGs are constructed via standard textile manufacturing techniques such as weaving (plain, satin, 3D weaves), knitting (weft, warp, interlock), and non-woven bonding. This level of integration offers unparalleled advantages in wearability, including breathability, drapability, and comfort. The fabric structure—determined by the weave/knit pattern, density, and yarn interlacing points—macroscopically governs the effective contact area, deformation mode, and thus the triboelectric output and sensing characteristics. For example, a woven structure provides high dimensional stability for pressure sensing, while a knitted structure offers high elasticity for strain sensing. The working principle operates at the macro-scale, involving programmable contact-separation between different fabric layers or within the fabric’s interlocking geometry under external pressure, stretch, or vibration, enabling energy harvesting and multi-modal sensing across large areas [75,76].

The hierarchical progression from fiber to yarn to fabric is not merely a matter of scale but a deliberate engineering paradigm where material properties are sequentially translated and amplified through structural design. This architecture-centric framework provides the essential foundation for the subsequent discussion on how these engineered textiles are deployed for sensing specific physiological and biomechanical signals in smart healthcare and sports applications, which will be detailed in the following sections.

## 3. Smart Healthcare

While TENGs offer broad potential in energy harvesting and self-powered sensing, their implementation in textile-based architectures presents distinct advantages for continuous, non-invasive health monitoring. Unlike rigid or semi-rigid configurations, T-TENGs inherently possess characteristics—such as flexibility, breathability, comfort, and conformability to dynamic body contours—that are crucial for long-term wearability. These properties make T-TENGs particularly well-suited for detecting subtle physiological signals including pulse, respiratory patterns, and sleep-related movements. The intimate and unrestrictive skin-garment interface enabled by textiles ensures reliable signal acquisition without compromising user comfort or natural activity, which is fundamental for practical healthcare applications. The following subsections detail how specific material strategies and structural designs of T-TENGs are leveraged to monitor these key physiological indicators effectively.

In the field of personal health management, smart healthcare has changed from passive treatment to proactive prevention through integrated technologies for continuous monitoring of pulse, respiration, and sleep. Utilizing sensors such as photoelectric, pressure, and motion detectors in wearable devices, it enables real-time and unobtrusive capture of multidimensional physiological data, including heart rate, respiratory frequency, and sleep architecture [77,78,79]. Empowered by artificial intelligence analytics, these technologies not only support daily health tracking but also facilitate early detection of issues such as cardiovascular abnormalities (e.g., arrhythmias), respiratory disorders (e.g., sleep apnea), and sleep quality disturbances. This allows for personalized risk warnings and health insights, while also providing critical data support for remote healthcare and chronic disease management [80,81,82,83]. Ultimately, it contributes to building an all-day, intelligent health protection ecosystem.

### 3.1. Pulse Monitoring

Pulse signals are vascular pressure fluctuations caused by periodic cardiac contractions and, as an important physiological signal of the human body, can reflect heart rate, vascular elasticity, and the overall state of the cardiovascular system. Pulse monitoring plays a critical role in early cardiovascular disease screening and long-term health management, making efficient and accurate pulse monitoring highly significant for smart healthcare and wearable health monitoring [84,85]. With the development of smart healthcare and wearable health monitoring, pulse signal acquisition has gradually shifted from intermittent, clinical measurements to continuous, wearable, and low-power monitoring modes. However, traditional pulse monitoring technologies such as photoplethysmography (PPG) or piezoresistive/capacitive pressure sensors usually rely on external power sources and are susceptible to motion artifacts, ambient light variations, and long-term wearing instability, which limits their application in long-term health monitoring scenarios [84,85]. TENGs utilize triboelectric and electrostatic induction effects to directly convert low-frequency mechanical stimuli generated by the human body into electrical signals, making them particularly suitable for detecting physiological signals such as pulse waves that are low in amplitude, low in frequency, and periodic [84]. When T-TENGs are applied to pulse-sensitive regions such as the wrist, fingertips, or neck, the periodic arterial pulsation induces slight deformation of the fabric structure, leading to changes in charge distribution at the triboelectric interface and driving electron flow in the external circuit, thereby generating electrical signals closely correlated with the pulse waveform. TENGs have four fundamental working modes: contact–separation mode, lateral sliding mode, single-electrode mode, and freestanding triboelectric-layer mode. Pulse monitoring has been one of the most extensively investigated applications of tri-boelectric nanogenerators, in which flexible thin-film devices have been widely adopted owing to their well-defined material systems, controllable micro-/nanostructures, and high pressure sensitivity. These characteristics enable thin-film TENGs to effectively resolve weak arterial pulse waveforms and therefore establish a solid technical foundation for triboelectric pulse sensing [84].

In flexible thin-film TENG-based pulse sensors, researchers typically select polymer materials with pronounced differences in the triboelectric series (such as PTFE, FEP, PDMS, and PVDF) as triboelectric layers, combined with flexible metal or conductive nanomaterial electrodes to enhance interfacial charge transfer efficiency [84,86,87]. On this basis, the introduction of micro/nanostructural engineering approaches (such as nanowire arrays, bilayer nanostructures, and biomimetic tympanic-membrane-like structures) can significantly amplify deformation induced by weak arterial pressure, thereby improving device sensitivity and signal-to-noise ratio in the low-pressure regime [84,85,87].

**Figure 1:**
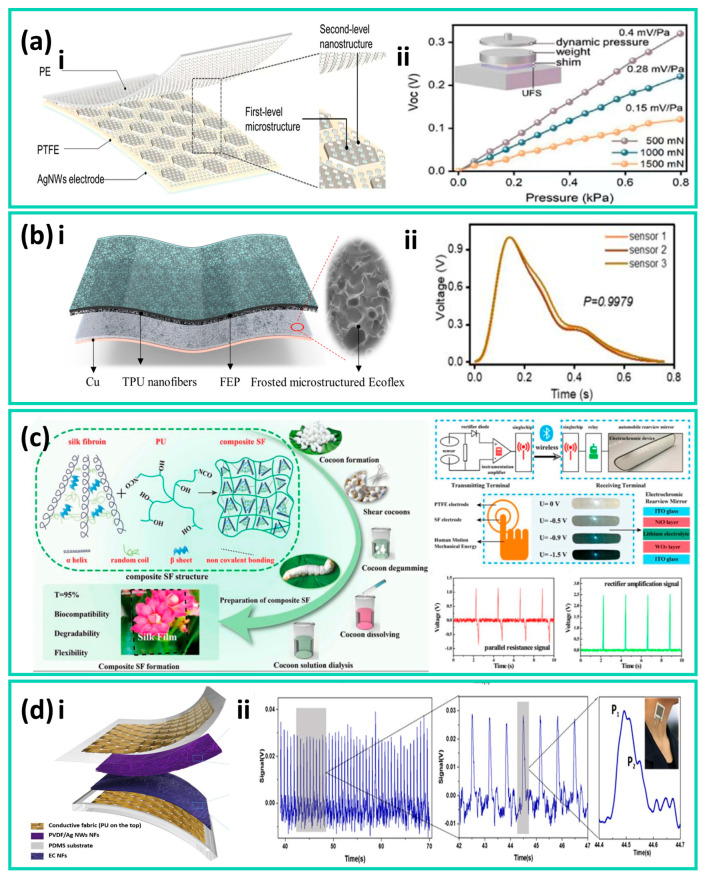
(**a**) (i) Schematic illustration of the ultraflexible sensor (UFS). (ii) Dynamic pressure response of the UFS under static conditions at applied forces of 500, 1000, and 1500 mN. Adapted with permission from Ref. [84]. Copyright 2003 John Wiley and Sons. (**b**) A flexible sensing array composed of an Ecoflex film and a TPU nanofiber composite layer. (i) Structural schematic of a single sensor unit. (ii) Measured signals of pulse monitoring at multiple localized positions on the wrist. Adapted from Ref. [88]. (**c**) Fabrication process of the TENG for smart fabric (SF) applications and its corresponding response signals. Adapted with permission from Ref. [90]. Copyright 2021 American Chemical Society. (**d**) Structure, sensitivity, and pulse wave experimental measurements of a triboelectric all-fiber-structured pressure sensor for pulse wave monitoring. (i) Design schematic of the textile-based sensor. (ii) Real-time output signals when the sensor fabric is worn on the wrist. Adapted with permission from Ref. [84]. Copyright 2020 American Chemical Society.

In 2021, an ultra-thin flexible sensor (UFS) was developed, featuring a single-electrode and a double-layer nanostructure design, composed of a flexible polymer triboelectric layer with nanoscale surface microstructures laminated with a flexible conductive electrode layer. The triboelectric layer was fabricated via a nanomold replication process, while the electrode layer was deposited on a flexible substrate using physical deposition methods. This ultrathin structure can conform closely to the skin, exhibiting a pressure sensitivity of approximately 0.15 mV·Pa^−1^ within the 0–0.8 kPa pressure range, and can stably capture pulse wave signals through brief fingertip contact, as shown in Figure 1a [84]. However, such devices primarily rely on laminated polymer films and metal electrodes, representing a typical non-textile flexible device system. In real continuous-wear scenarios, they face limitations in breathability, washability, and scalable manufacturing. To address this, researchers have further improved the stability and reliability of low-frequency pulse signal sensing by integrating material composites and elastic structural designs while retaining the high sensitivity advantage of thin-film devices.

Wang et al. developed a flexible sensing array based on a frosted microstructured Ecoflex film and TPU Nanofibers for Epidermal Pulse Wave Monitoring, capable of clearly resolving the main peak and reflected pulse waves at the wrist and neck, as shown in Figure 1b. The Ecoflex elastomer was prepared by casting and curing on a frosted template, forming randomly distributed microscale rough structures on its surface, while the TPU nanofiber layer was fabricated via electrospinning. The two layers were then laminated and integrated with flexible electrodes to construct the sensing array. The single sensor exhibited a sensitivity of 0.14 mV·Pa^−1^, a response time of 22 ms, a low-frequency range of 1–23 Hz, and stability up to 7000 cycles, demonstrating the potential of microstructured elastomers for pulse monitoring applications [88]. Furthermore, introducing functional fillers into a flexible matrix to modulate the intrinsic mechanical and interfacial properties of materials has also been demonstrated as an effective performance-enhancement strategy.

In terms of structural innovation, Zhao et al. proposed the in situ air gap-generation method, to prepare a no-spacer TENG (NSTENG), in which a controllable air gap is formed in situ between triboelectric layers through structural design during fabrication, eliminating the need for conventional spacers and enabling a spacer-free contact–separation TENG configuration. Compared with conventional spacer-supported structures, this design effectively avoids local stress concentration induced by spacers, allowing the device to generate larger displacement under the same applied pressure and achieve more uniform stress–strain distribution. In rat heart rate monitoring experiments, the heart rate detection accuracy reached as high as 99.73%, demonstrating excellent reliability in cardiovascular monitoring [89].

Based on a similar strategy, Jia et al. developed an EPGS-TENG that employs a gas layer as the supporting structure and an Ecoflex–PVDF composite as the negative triboelectric layer. The gas layer is a sealed air chamber formed by in situ encapsulation between the upper and lower flexible triboelectric layers, which utilizes gas compressibility to provide uniform elastic support under external force and drives rapid separation of the triboelectric layers upon release, thereby enabling stable, high-displacement contact–separation motion without spacers. The device exhibits a power output of 121 µW, a pressure sensitivity of 7.57 V·N^−1^, temperature tunability from 20° to 40°, and an angular response capability of 374%, making it suitable for pulse signal acquisition in complex human-body environments [89].

However, pulse monitoring in real-world wearable scenarios places additional requirements on breathability, conformability, mechanical robustness, and long-term wearing comfort, which are not fully addressed by conventional thin-film configurations. In this context, textile-based triboelectric nanogenerators, which integrate triboelectric sensing functions directly into fibers, yarns, and fabric architectures, have emerged as a promising alternative, offering a more balanced combination of sensing performance and wearability.

By contrast, T-TENGs place greater emphasis on stability and comfort under real-world wearing conditions. Such devices are typically fabricated using nylon or PVDF yarns and conductive fibers through braiding, knitting, or weaving to form integrated sensing structures, offering good breathability, flexibility, and washability, and are therefore suitable for continuous pulse monitoring [53,91,92].

In fiber-based devices, Graham et al. fabricated positive triboelectric poly(vinyl alcohol) (PVA) fibers and negative triboelectric polycaprolactone (PCL) fibers via electrospinning, and constructed multi-point fiber contact interfaces through interlayer alignment or crossed fiber bundle stacking to form a fiber–fiber contact–separation triboelectric structure, thereby realizing a totally biocompatible F-TENG. The device exhibits good consistency in output performance and can be applied to various medical monitoring scenarios, primarily targeting epidermal pulse detection and weak physiological vibration sensing, while also serving as a self-powered unit for low-power medical electronic systems [90]. This type of research verifies the feasibility of T-TENGs at the material and fiber levels, but their degree of functional integration still requires further enhancement at the textile scale.

Building upon this fiber-level feasibility, researchers have further explored textile-scale structural configurations to enhance pressure responsiveness under subtle physiological stimuli. In this context, Ding et al. proposed a triboelectric all-fiber structured pressure sensor via a facile electrospinning technique in 2020. This device employs polyvinyl chloride/nitrogen nanowire composite nanofiber membranes and ethyl cellulose nanofiber membranes as triboelectric layers, fabricated via electrospinning and sandwiched between two layers of conductive fabrics to form a vertical contact–separation structure. By introducing hierarchical surface roughness on the nanofibers through electrospinning, the deformation response of the textile to subtle pressure variations was significantly enhanced. The sensor achieved sensitivities of 1.67 mV Pa^−1^ and 0.2 mV Pa^−1^ in the pressure ranges of 0–3 kPa and 3–32 kPa, respectively, and maintained stable output after 7200 continuous cycles, as shown in Figure 1d. The device can clearly resolve pulse waveforms, demonstrating its reliability for long-term cardiovascular monitoring [84].

**Figure 2:**
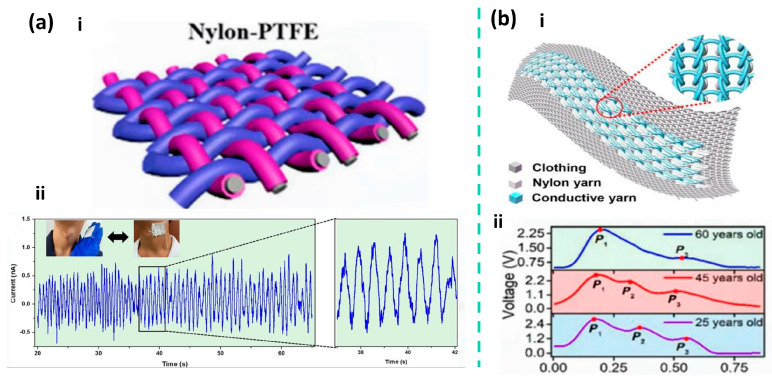
(**a**) Structure, sensitivity, and experimental pulse wave measurements of a self-powered triboelectric sensing textile. (i) Schematic illustration of the self-powered triboelectric sensing textile. (ii) Real-time detection of pulse waves and amplified carotid artery pulse signals using the sensing textile. Adapted with permission from Ref. [84]. Copyright 2020 American Chemical Society. (**b**) (i) Schematic illustration of the triboelectric all-textile sensor array (TATSA). (ii) Real-time output voltage when the TATSA is placed on the arterial region of a female wrist. Adapted with permission from Ref. [86]. Copyright 2020 American Association for the Advancement of Science.

In terms of integrated textile structural design, Li et al. developed a triboelectric sensing textile composed entirely of fibers, with twisted helical core–shell yarns as the key structural element. In this design, conductive fibers act as the core yarn to provide charge transport and mechanical support, while triboelectrically active fibers serve as the shell yarn helically wrapped around the core, enabling structural integration of the triboelectric and conductive layers at the fiber scale and direct participation in textile fabrication. This design eliminates the need for additional adhesives or metal film encapsulation, allowing the device to maintain excellent mechanical strength while offering good breathability and wearing comfort. The sensing textile achieves sensitivities of 1.33 V kPa^−1^ and 0.32 V kPa^−1^ in the pressure ranges of 1.95–3.13 kPa and 3.20–4.61 kPa, respectively, and maintains stable sensing performance even after 4200 cycles and continuous washing for 4 h. During carotid artery pulse monitoring, the textile can continuously and clearly record pulse wave signals, as shown in Figure 2a, demonstrating its advantages for washable and long-term wearable pulse monitoring [84].

In addition to conventional woven structures, nonwoven fibrous architectures have emerged as effective structural pathways for the realization of fully textile-based triboelectric nanogenerators (T-TENGs). Kim et al. reported an all-textile structured TENG composed of a PVDF/MXene nanofiber membrane and an Ag@nylon 6,6 fiber membrane. In this device, a PVDF/MXene composite nanofiber membrane fabricated via electrospinning serves as the negative triboelectric layer, while a silver nanoparticle-coated nylon 6,6 fiber membrane (Ag@nylon 6,6) simultaneously functions as the positive triboelectric layer and the conductive electrode. The two fibrous textiles are structurally integrated in a face-to-face stacked configuration, enabling periodic fabric–fabric contact and separation under external mechanical stimulation, thereby forming an all-textile triboelectric structure operating in the contact–separation mode. The introduction of MXene significantly enhances the dielectric properties and surface charge trapping capability of the PVDF nanofiber membrane, thereby improving triboelectric output, while the Ag@nylon 6,6 fiber membrane provides stable electrical conduction and imparts antibacterial functionality to the device. Based on this fully textile-based structure, the integrated system can achieve stable pulse signal acquisition under practical wearing conditions and can be further extended to application scenarios such as intelligent human–machine interaction [93].

When a single fabric-based triboelectric unit cannot satisfy the demands of multi-parameter or multi-site synchronous monitoring, array-based integration becomes a key structural strategy for further enhancing information acquisition capability. Meng et al. and Fan et al, respectively, developed a textile-based sensor (TS) that has a stable bilayer structure constructed in the form of a flower and a washable T-TENG sensor array. In the flower-shaped T-TENG, flexible triboelectric films and conductive fabrics form the basic triboelectric units, which are structurally cut and radially arranged into petal-like shapes and integrated into arrays on a textile substrate, thereby significantly increasing the effective contact area and deformation consistency at the fabric scale. This sensor array achieves a sensitivity of up to 7.84 mV·Pa^−1^, a response time of approximately 20 ms, and supports wireless data transmission to mobile terminals [64]. In contrast, the washable T-TENG constructs a sensing array based on a knitted integration of conductive yarns and triboelectric yarns, exhibiting stable signal output together with excellent mechanical durability and washing stability. These array-based structures enable the orderly integration of multiple independent triboelectric sensing units at the textile scale, allowing each unit to respond simultaneously to external mechanical stimuli at different spatial locations. As a result, spatial discrimination and synchronous acquisition of physiological signals such as pulse and respiration are achieved, highlighting the advantages of T-TENGs in system-level integration for wearable physiological monitoring applications [64,93].

From the perspective of scalable manufacturing, a triboelectric sensor array based on a fully fiber-based weft-knitted structure, known as the triboelectric all-textile sensor array (TATSA), demonstrates excellent pulse monitoring performance. TATSA is a triboelectric sensor array composed entirely of textile yarns, employing stainless-steel fiber–core conductive yarns and commercial nylon yarns to form a highly compressible double-layer fabric with a large effective contact area via a full cardigan stitch structure. This T-TENG exhibits a sensitivity of approximately 7.84 mV·Pa^−1^ in pulse pressure sensing and maintains stable output after 10^5^ loading cycles, demonstrating good mechanical durability and long-term reliability. The array can be directly worn on typical monitoring sites such as the wrist, continuously acquiring clear and repeatable pulse waveforms in real application scenarios, as illustrated in Figure 2b [84,93]. Although T-TENGs generally exhibit lower ultimate sensitivity compared with micro- or nanostructured thin-film devices, they demonstrate a higher level of application maturity [53,85,91].

Overall, flexible thin-film TENGs and T-TENGs exhibit distinct emphases in structural design strategies and performance advantages for pulse monitoring applications, and their representative approaches and performance impacts can be summarized as shown in Table 2. Thin-film TENGs, benefiting from precisely controllable material compositions and layered or air-gap structures, generally demonstrate higher pressure sensitivity and signal resolution, making them suitable for high-precision detection of weak pulse waveforms; however, their breathability and long-term wearing comfort are relatively limited. In contrast, T-TENGs enhance flexibility, breathability, and mechanical durability through the optimization of fibers, yarns, and textile architectures while maintaining sufficient output performance, rendering them more suitable for continuous pulse monitoring in real wearable scenarios. Meanwhile, textile-scale and array-based structures exhibit clear advantages in output stability and scalability. In summary, future pulse monitoring devices for smart healthcare are expected to achieve synergistic optimization of performance and comfort by integrating the high sensitivity of thin-film TENGs with the wearable advantages of T-TENGs [53,84,86,92].

When assessing the translational potential of the devices discussed, a clear demarcation exists between laboratory proof-of-concept and systems nearing practical application. As shown in Table 3, most flexible thin-film TENG pulse sensors (e.g., [66,70,71]) are predominantly proof-of-concept demonstrations, optimized for maximal sensitivity under controlled conditions but lacking the necessary attributes (washability, long-term comfort, robust textile integration) for sustained wearable use. In contrast, advanced textile-integrated designs like the washable triboelectric sensing textile [66] and the knitted Triboelectric All-Textile Sensor Array (TATSA) [76] represent significant strides toward application-ready systems. Their focus on textile-process compatibility, mechanical durability over thousands of cycles, and stable performance after washing addresses key practical barriers, positioning them closer to prototype validation in real-world settings.

Despite promising progress, TENG-based pulse sensors face inherent trade-offs rooted in their design. Flexible thin-film TENGs achieve high sensitivity often at the expense of breathability, long-term wearing comfort, and washability, limiting their practicality for continuous use. Conversely, textile-configured T-TENGs prioritize wearability but generally exhibit lower ultimate sensitivity and can be susceptible to motion artifacts from fabric shear or slippage. A recurring limitation across both types is the difficulty in maintaining stable interfacial charge density under repetitive mechanical cycling and varying environmental humidity, which directly impacts signal-to-noise ratio and long-term reliability. Furthermore, the integration of high-sensitivity micro/nanostructures into scalable, robust textile manufacturing processes remains a significant materials engineering challenge. To address these challenges, future research should focus on hybrid approaches and material innovations. For thin-film devices, incorporating breathable nanofiber membranes or porous electrodes could reconcile sensitivity with comfort. Textile-based sensors may be improved through surface-engineered yarns (e.g., core–shell structures) and optimized fabric patterns to enhance sensitivity and reduce motion artifacts. Stable charge density under humidity could be achieved via hydrophobic nanocomposite coatings or sealed breathable encapsulation. Finally, scalable integration of micro/nanostructures calls for adopting textile-compatible techniques like roll-to-roll printing or pre-fiber functionalization to bridge lab designs and industrial production.

### 3.2. Sleep Monitoring

In sleep monitoring applications, T-TENGs primarily function as integrated self-powered sensing platforms. This dual functionality stems from their inherent working principle: they directly convert low-frequency biomechanical energy from sleep-related movements—such as body pressure shifts, respiratory-induced fabric deformation, and limb micromovements—into analyzable electrical signals without requiring an external power source. Unlike systems where sensing and powering units are separate, T-TENGs achieve simultaneous energy harvesting and signal transduction. This integrated approach is particularly advantageous for constructing unobtrusive, long-term wearable or bedding-integrated systems. Crucially, the physiological signals they capture are inherently interconnected. For instance, cardiopulmonary coupling—linking heart rate variability (associated with pulse signals, Section 3.1) and respiratory rhythm (detailed in Section 3.3)—is a key metric for sleep staging. Furthermore, body movement patterns and posture changes, detected via pressure distribution, often correlate with respiratory events or sleep disorders. Therefore, T-TENGs enable the correlative acquisition of multi-parameter data, providing a comprehensive foundation for sleep architecture analysis and disorder screening.

Sleep monitoring is a crucial approach for assessing neurological function, diagnosing sleep disorders, and maintaining overall health [94,95,96,97,98]. Although traditional polysomnography (PSG) is regarded as the clinical “gold standard,” its reliance on numerous surface electrodes and wired connections causes subject discomfort, disturbs natural sleep architecture, and hinders long-term home-based monitoring. In recent years, non-invasive wearable devices based on photoplethysmography (PPG), accelerometers, and heart-rate variability analysis have gained popularity, significantly improving accessibility and user experience [99]. However, existing technologies still face several inherent limitations: the mechanical mismatch between rigid electronic components and the skin interface compromises wearing comfort and signal stability; battery dependence restricts long-term continuous operation and further miniaturization; and most sensors are single-functional, making it difficult to simultaneously capture multimodal sleep-related physiological parameters such as respiratory rhythm, body micro-movements, and electrodermal activity. Against this background, T-TENGs offer a transformative pathway toward constructing a new generation of “unperceivable” smart sleep-monitoring systems, owing to their flexibility, self-powering capability, broad material choices, and high sensitivity to various mechanical stimuli. By integrating triboelectric functional fibers into bed sheets, pillowcases, or sleepwear, TENGs can convert subtle motions, breathing undulations, and even cardiac vibrations during sleep into electrical signals without the need for batteries, enabling all-night, comfortable, and multi-parameter sleep-quality assessment and disorder screening [96,100,101,102,103].

**Figure 3:**
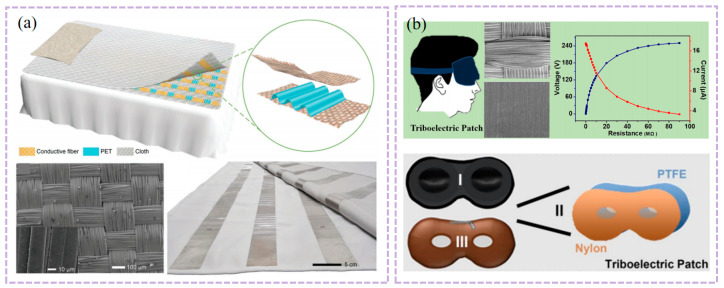
(**a**) Structure design of the TENG based self-powered smart textile. Adapted with permission from Ref. [104]. Copyright 2017 John Wiley and Sons. (**b**) Simulated diagram and Schematic diagram of the triboelectric patch components. Blue and red lines are maximum output voltage and current versus different load resistances. Adapted with permission from Ref. [105]. Copyright 2022 American Chemical Society.

This study presents a large-scale, washable smart textile based on a TENG array for self-powered sleep monitoring [104]. As shown in Figure 3a, the textile is constructed from conductive Ag-coated fibers arranged in perpendicular rows and columns, sandwiching a wavy-structured polyethylene terephthalate (PET) film as the triboelectric layer. This unique structural design enhances pressure sensitivity by increasing the effective contact area under compression. The device demonstrates a high pressure sensitivity of 0.77 V Pa^−1^, a fast response time of <80 ms, and excellent mechanical durability, maintaining stable electrical output over 5000 cycles. Here, the TENG array functions primarily as a self-powered pressure sensing platform, converting body pressure and posture changes into mappable electrical signals for sleep tracking and quality assessment. Moreover, it retains performance after repeated washing, confirming its robustness for daily use. Integrated with a multichannel data acquisition and wireless transmission system, the textile enables real-time mapping of body pressure distribution, sleep posture tracking, and sleep quality assessment with high spatial and temporal resolution. It also functions as a self-powered alert system for falls, demonstrating its potential for reliable, non-invasive, and continuous sleep monitoring in clinical and home settings.

This study reports a self-powered triboelectric patch based on Maxwell’s displacement current for human energy harvesting and eye-movement monitoring during sleep [105]. As shown in Figure 3b, the patch employs inductively coupled plasma (ICP)-etched polytetrafluoroethylene (PTFE) and nylon fabric as triboelectric layers, with a three-layer structure: a skin-friendly 3D inner patch, the ICP-processed friction layers, and an outer layer that can be filled with functional materials such as cassia seeds. This device exemplifies the dual role of TENGs: as an energy harvester (powering small electronics) and as a specific physiological signal transducer (converting eyelid motion into detectable voltages for REM sleep identification). The nanostructured surfaces enhance the contact area and triboelectric charge density, yielding an open-circuit voltage up to 385 V and a short-circuit current of 62 μA under periodic contact-separation motion. For eye-movement monitoring, the patch can generate a detectable voltage signal (e.g., 80 V at a 20 mm vertical distance) corresponding to eyelid motion, enabling the distinction of rapid-eye-movement (REM) sleep stages when combined with cardiopulmonary coupling analysis. The patch maintains stable electrostatic fields after repeated washing, with surface potential changing only from −3.36 kV to −3.33 kV, demonstrating excellent durability and suitability for daily wearable use. This work provides a comfortable, washable, and self-powered approach for continuous eye-movement tracking, offering potential for low-cost, non-invasive sleep-stage monitoring and early screening of sleep-related disorders.

The maturity of T-TENGs for sleep monitoring varies significantly. The large-scale smart textile array [87] exemplifies a system-level prototype that integrates sensing, data acquisition, and wireless transmission, demonstrating functionality in a realistic scenario (bedsheet) and addressing washability—a critical step beyond simple lab validation. The triboelectric patch for eye movement [88], while innovative in its self-powered sensing approach, remains primarily a specialized proof-of-concept for a specific physiological signal (eye movement) and requires further integration with comprehensive sleep staging algorithms and validation against clinical polysomnography to be considered application-ready.

The deployment of T-TENGs for sleep monitoring reveals several systemic constraints. Firstly, the low-frequency, low-amplitude nature of sleep movements often results in weak electrical signals, demanding exceptionally high sensor sensitivity and sophisticated signal processing to distinguish physiological events from noise. Secondly, the pressure distribution across a large-area textile sensor array can be non-uniform, leading to inaccurate body posture mapping if calibration is inadequate. A key structural limitation is the compromise between sensor density (for spatial resolution) and system complexity (wiring, data channels), which affects scalability and cost. Moreover, the long-term mechanical durability of triboelectric layers under nightly compression cycles and the effects of bedding materials on triboelectric output require further investigation for reliable commercialization.

As shown in Table 4, T-TENGs offer a unique “sensing-powering” integrated pathway for sleep monitoring. Unlike optical or inertial sensors that rely on external power, TENGs directly convert mechanical stimuli during sleep into self-powered signals, enabling comfortable, long-term, and multi-parameter monitoring. The signals acquired—encompassing body pressure (related to posture and movement), respiratory-induced motions (linked to Section 3.3), and even subtle cardioballistic vibrations (associated with pulse, Section 3.1)—can be synergistically analyzed to provide a holistic view of sleep physiology. Current research has demonstrated its potential in terms of sensitivity, durability, and system integration. Future advancement hinges on: enhancing signal processing to accurately identify specific sleep events by leveraging the fusion of multiple physiological signals; promoting deeper functional integration with other flexible sensors for complementary data; and establishing reliable evaluation standards through rigorous clinical studies that validate the correlations between T-TENG-derived multi-parameter signals and traditional polysomnography metrics.

**Table 4:** Performance summary of representative T-TENGs for sleep monitoring.

Representative Type	Positive Tribo-Material	Negative Tribo-Material	Electrical Output Performance	Stability (Washability/Durability)	Ref.
Single-Electrode Mode Fabric T-TENG	Human skin, Nylon fabric, Cotton fabric	PTFE nanofiber membrane, Silicone rubber	Peak Voc: Up to ~1050 V	Washable: T-TENG sewn with nanofiber PTFE membrane shows stable performance after washing.	[53]
Core–Shell Structured Nanoyarn TENG	Silver-coated nylon yarn (Conductive core)	PVDF-TrFE/Cs_3_Bi_2_Cl_9_ composite sheath layer	Sensitivity: 3.64 V/kPa Durability: >50,000 cycles	Wear-resistant & Durable: Integrated core–shell structure with high mechanical stability.	[106]
Bed Sheet/Mattress-Integrated T-TENG Array	Fabric (e.g., Cotton, Polyester) or Composite conductive fabric	PDMS, PTFE, Silicon-based elastomer	Output depends on array area & pressure; Aimed at achieving sensing functionality.	Scalability & Washability: Some are designed as large-scale, washable smart bedsheets.	[104]
Smart Pillow T-TENG Array	Flexible electrodes & breathable tribo-materials (e.g., Nylon mesh)	Porous silicone, FEP film, etc.	For monitoring head pressure distribution & movement; Output correlates with pressure location and magnitude.	Breathability & Comfort: Design emphasizes flexibility and breathability, key for long-term contact monitoring.	[107]

### 3.3. Respiratory Monitoring

T-TENGs, as quintessential textile-based intelligent devices, represent a pivotal technological direction in the field of smart healthcare respiratory monitoring. The construction of reliable signal acquisition hardware through material functionalization and hierarchical structural design forms the foundation for monitoring capabilities and has been a central research focus in recent years. To enhance the triboelectric effect and charge trapping capacity at the textile interface, current research primarily focuses on three aspects: material functionalization strategies, structural innovation based on fibers, yarns, or fabrics, and multi-mechanism synergy. These efforts aim to improve the electrical responsiveness to subtle respiratory signals.

#### 3.3.1. Material Functionalization and Layered Structure Design

**Figure 4:**
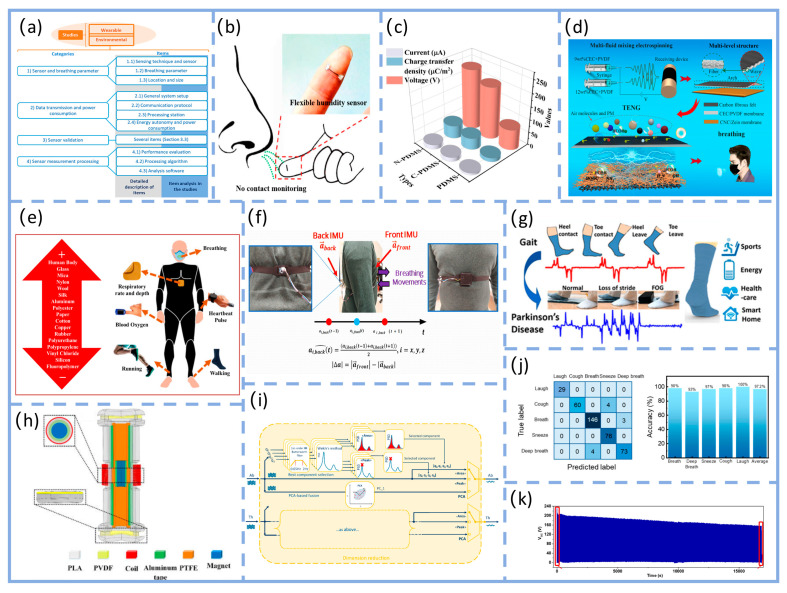
Structural principles, performance comparisons, and signal processing methods for respiratory monitoring technologies. (**a**) Respiratory monitoring system analysis structure. Adapted from Ref. [121]. (**b**) Respiratory monitoring schematic. Adapted from Ref. [108]. (**c**) Comparison of TENG output performance values based on PDMS, C-CdS/PDMS, and N-CdS/PDMS, including output voltage, current, and charge transfer density. Adapted from Ref. [122]. (**d**) Schematic of cellulose-based TENG fabrication with multilayer structure. Adapted from Ref. [110]. (**e**) Principle of skin-contact TENG. Adapted from Ref. [123]. (**f**) Schematic illustrating the working principle of an advanced dual-inertia chest strap; red arrows indicate inertial sensor positions, while purple arrows represent chest movement during respiration. Adapted from Ref. [120]. (**g**) Self-powered cotton socks created using piezoelectric and TENGs. Adapted with permission from Refs. [111,124]. Copyright 2019 American Chemical Society. (**h**) Three-dimensional structure of a piezoelectric-electromagnetic-triboelectric hybrid energy harvester. Adapted from Ref. [114]. (**i**) Detailed downscaling blocks. Starting from the four components [q0, q1, q2, q3] of each quaternion (abdomen: Ab and thorax: Th), three methods are applied to obtain a single fractional signal: two methods based on optimal quaternion component selection (“Region” and “Peak”), and one method based on fusing the four components via principal component analysis (PCA). Adapted from Ref. [117]. (**j**) Demonstration of the wireless real-time respiratory monitoring system. Confusion matrix and classification accuracy for five respiratory pattern recognitions. Adapted from Ref. [118]. (**k**) Output stability of WF-TENG. Output voltage during approximately 5 h of continuous WF-TENG operation. Adapted from Ref. [119].

As illustrated in Figure 4a, Chen et al.’s work provides a representative example. Their study did not solely pursue power generation efficiency in material selection but instead established a triple balance of “biocompatibility-signal capture capability-environmental adaptability.” Through hierarchical structural design, they achieved synergistic enhancement of these three core performance metrics. For wear comfort, a biocompatible polydimethylsiloxane (PDMS) was used as the flexible substrate. After plasma activation treatment, the substrate’s surface contact angle decreased significantly from ~102.6° to ~26.5°, greatly enhancing interfacial wettability and adhesion. Simultaneously, the non-contact design enables respiratory monitoring from a 3 cm distance, completely eliminating the discomfort associated with traditional skin-contact monitoring and significantly optimizing wearability. This unit features micro-nano-scale irregular protrusions that not only enhance interfacial bonding strength but also confer exceptional flexibility to the sensor. It maintains stable performance after 500 bending cycles, effectively resisting mechanical deformation interference during wear. For signal consistency, the micro/nano-scale irregular protrusions significantly increase the contact area between the sensing unit and water molecules. Combined with the construction of a three-dimensional conductive network, this reduces the sensor response time to 0.35 h, enabling precise capture of minute humidity changes in respiratory rhythms. The sensor demonstrates high repeatability and long-term stability across a 20–90% relative humidity range [108].

Another study further enhanced performance through biomimetic design, as shown in Figure 4b, a composite triboelectric couple was constructed using fish gelatin (FG, derived from fish connective tissue) and polydimethylsiloxane (PDMS, modified with dopamine and fluorosilane) as the friction layer, forming a high-performance FG-TENG with blade-inspired microstructures. Through hierarchical structural design, this device exhibits outstanding power generation performance: peak open-circuit voltage reaches 320 V, short-circuit current is 0.80 μA, and output power density is significantly enhanced—capable of reliably driving miniature electronic devices. In sensing applications, when attached to the human body, it can monitor movement states such as walking, running, jumping, leg swing, and vocal cord vibration. Signal consistency is demonstrated by a coefficient of variation in output voltage of only 3.2% over 100 consecutive cycles, with motion recognition response time ≤ 10 ms, enabling precise tracking of physiological activities. Benefiting from the biocompatibility and degradability of FG, the device ensures high output performance while offering wearable comfort (skin irritation tests show no redness, swelling, or allergic reactions) and mechanical stability (performance retention rate ≥ 90% after 1000 bending cycles). With a device thickness of only 80 μm, it boasts good biocompatibility, non-toxicity and non-irritability, conforms to the body’s contours without noticeable foreign body sensations, exhibits excellent mechanical stability that can withstand 1000 bending cycles at a radius of 2 cm, and features reliable output stability [109]. This makes it particularly suitable for long-term health monitoring in sensitive populations. This demonstrates that combining hierarchical structure with material biocompatibility is an effective approach to achieving both high performance and high comfort. Similarly, cellulose-based TENGs fabricated via multi-fluid electrospinning (comprising cellulose nanocrystal/zein (CNC/zein) and cyanethylcellulose/polyvinylidene fluoride (CEC/PVDF) membranes) leverage biocompatibility advantages while achieving multifunctional synergy between respiration sensing and air purification.

Cellulose-based TENGs demonstrate novel approaches to functional integration, as illustrated in Figure 4c, this device utilizes cellulose nanocrystal/corn zein (CNC/zein) membranes and cellulose ethyl cellulose/polyvinylidene fluoride (CEC/PVDF) membranes as core materials. Through multi-fluid electrospinning, it constructs a fiber-wave-arch three-tiered structure and multi-fluid electrospinning, it constructs a fiber-wave-arch three-tiered structure (microscopically featuring high-roughness nanofibers with nanospheres and pores, macroscopically forming regular parallel waves and spontaneously retracted arches). At 6 wt% (mass fraction), the CEC/PVDF system exhibits a short-circuit current of 3.30 µA and an open-circuit voltage of 10.01 V. At 12 wt%, CEC/PVDF achieves 98.84% filtration efficiency against PM particles, while CNC/zein demonstrates 92% adsorption efficiency at 0.25 mg/m^3^ formaldehyde concentration. This approach eliminates external power constraints while reducing infection risks through air purification, achieving seamless integration of biocompatibility, signal stability, and clinical utility [110]. This case study further expands the functional boundaries of textile-based T-TENGs, demonstrating that through synergistic textile engineering of structural and material design, a transition from single-function monitoring to an integrated “monitoring-protection” system can be achieved.

#### 3.3.2. Multi-Mechanism Hybrid Energy Harvesting and Signal Enhancement

Single-TENG mechanisms face limitations in capturing low-frequency, weak physiological signals. To address this, researchers integrated additional energy harvesting mechanisms—such as piezoelectric (PEG) and electromagnetic (EMG)—to construct hybrid systems that synergistically enhance performance.

As illustrated in Figure 4f, PTNG employs polymer materials—particularly those with distinct triboelectric polarities—as the core functional components for both TENG and PENG. Structurally, it achieves synergistic energy harvesting and signal conversion through dual-mechanism integration. Application-wise, it systematically optimizes energy harvesting and signal conversion efficiency for low-frequency (0–15 Hz), small-amplitude physiological movements, making it particularly suited for long-term monitoring of weak physiological signals like respiration [111]. Mariello et al. proposed that intra-domain hybridization with physical integration—such as embedding piezoelectric nanofillers like barium titanate (BaTiO_3_) into triboelectric polymer matrices like polydimethylsiloxane (PDMS)—can enhance charge generation and transport through intermaterial synergies. As illustrated in Figure 4d, the composite system formed by piezoelectric nanoparticle fillers and triboelectric polymers eliminates the need for additional electrodes. Application-wise, the enhanced charge generation and transport efficiency significantly optimizes response sensitivity to low-amplitude mechanical disturbances, aligning with TENG’s requirement for capturing minute thoracic deformation signals in respiratory monitoring [112]. This design logic aligns closely with the “high sensitivity-low intrusiveness” requirements for respiratory sensors, providing clear theoretical and experimental guidance for structural optimization of PTNG in respiratory signal capture. It is particularly significant for enhancing signal consistency in dynamic scenarios. Haroun et al.’s research further corroborates this logic. Their study on low-frequency vibration scenarios demonstrates that hybrid architectures combining TENG with piezoelectric andelectromagnetic technologies can achieve dual synergistic enhancement in energy harvesting and vibration sensing. This approach not only improves energy conversion efficiency for low-amplitude vibrations through multi-mechanism coordination but also optimizes vibration signal capture accuracy. This aligns perfectly with the low-frequency (0.2–0.33 Hz) and small-amplitude characteristics of respiratory motion, providing cross-scenario experimental support for designing and optimizing hybrid T-TENG structures for respiratory monitoring [113].

Research by Haroun et al. and Tang’s team further validates the hybrid architecture’s advantages. As shown in Figure 4g, the non-resonant piezoelectric-electromagnetic-triboelectric hybrid energy harvester developed by Tang et al. significantly expands the application boundaries of low-frequency human motion energy harvesting at the performance, scenario, and system levels through multi-mechanism synergy and system integration. At the performance level, this device ingeniously integrates piezoelectric (PEG), electromotive (EMG), and triboelectric (TENG) mechanisms, overcoming the limitations of traditional single-TENG devices—namely, low output current and high internal resistance at low frequencies. Experiments demonstrate that under low-frequency conditions (4 Hz) simulating human motion, its PEG, EMG, and TENG units deliver maximum power outputs of up to 26.17 mW, 87.1 mW, and 63 μW, respectively, achieving milliwatt-level total energy output sufficient to power ultra-low-power sensors. In application scenarios, the device demonstrates excellent wearability, operating stably across diverse movement postures including wrist, hand, calf, and waist motions. The TENG unit achieves peak-to-peak output voltages of 140–220 V, proving its broad adaptability to complex, non-periodic human movements. Ultimately, at the system level, the research successfully established a complete closed-loop from energy harvesting to wireless transmission: energy output from the hybrid collector is efficiently stored via management circuits, enabling it to power a wireless temperature and humidity sensor module. This module can be activated via Bluetooth within approximately 10 s and continuously transmit data for up to 18 s, validating its practical potential as the core energy source for self-powered systems. Thus, this work not only enhances low-frequency energy harvesting efficiency through a hybrid architecture but also advances the application frontier of energy harvesting technology from laboratory prototypes to practical wearable systems supporting real-time sensing and wireless communication. This is achieved through multi-site wearable validation and system-level functional demonstrations, laying a solid technical foundation for developing self-powered clinical monitoring solutions [114]. The triboelectric-electromagnetic hybrid nanogenerator synchronously excites three energy harvesting units via a moving magnet, leveraging piezoelectric, electromagnetic, and triboelectric mechanisms. The electromagnetic unit delivers high current output, while the piezoelectric and triboelectric units contribute high voltage characteristics. This synergy achieves significant output enhancement under low-frequency (4 Hz) excitation, with the triboelectric unit reaching peak voltages of 150 V. The system stably adapts to human motion energy harvesting across multiple body regions, including the wrist and calf. This design philosophy aligns exceptionally well with the low-frequency, small-amplitude characteristics of respiratory motion. This not only validates the hybrid architecture’s advantage in capturing weak mechanical signals but also establishes a new reference for “multi-mechanism synergistic signal amplification” in T-TENG respiratory monitoring. It holds particular significance for enhancing the signal-to-noise ratio of respiratory signals in dynamic scenarios.

Beyond the hybrid architecture, a binary hybrid strategy combining triboelectric and electromagnetic mechanisms also demonstrates unique advantages for respiratory monitoring—as demonstrated by Wu et al.’s miniaturized high-performance hybrid nanogenerator (MHP-HNG). As illustrated in Figure 4h, this device achieves in-phase vibration by coupling TENG with EMG. Following optimization of the grid structure friction layer and Halbach magnet array, it achieves an output voltage of 14.14 V and with a peak power of 49 mW. The device dimensions are only 60 mm × 40 mm × 10 mm, meeting wearable convenience requirements [115].

This hybrid design inherently aligns with the low-frequency, small-amplitude characteristics of respiratory motion. By employing “multi-mechanism synergistic signal amplification,” it significantly enhances the ability to capture weak mechanical signals in dynamic scenarios, which is crucial for improving the signal-to-noise ratio of respiratory signals.

#### 3.3.3. Signal Processing, Algorithm Enhancement, and Performance Validation

Beyond hardware optimization, signal processing and algorithms play a critical role in enhancing the reliability and intelligence of the TENG respiratory monitoring system.

Regarding signal stability, factors like woven structures significantly impact contact area and output consistency, particularly in breathing patterns involving body movement where signals are susceptible to interference [116]. The PCA fusion method combines the four components of quaternions into a single respiratory signal with maximum variance through principal component analysis, thereby maintaining stable signal extraction performance across different postures, as illustrated in Figure 4i. This provides a reference for designing T-TENG differential structures with interference resistance and multi-scenario adaptability [117].

The core of performance optimization lies not in maximizing a single metric, but in achieving a quantitative balance among multiple factors. Improvements in sensitivity, response time, and accuracy depend on the coordinated regulation of materials, structure, environment, and algorithms. The practical value of this balance must be demonstrated through comprehensive validation [118]. Compared to traditional rigid or non-textile sensors (e.g., piezoelectric or capacitive sensors), it demonstrates performance advantages under dynamic wear conditions. For instance, the literature proposes textile-based T-TENG as a self-powered pressure sensor for physiological signal monitoring, whereas most conventional sensors require external power sources and offer inferior wear comfort. After structural optimization, its error can be reduced to ±0.3 breaths per minute, quantitatively validating the enhancement of signal stability and measurement accuracy through textile structural design. Furthermore, this optimized performance meets general standards for clinical respiratory rate monitoring, enabling continuous, non-invasive monitoring while maintaining reliability and consistency within typical physiological ranges (e.g., adult resting respiratory rate of 12–20 breaths/min). This satisfies the dual requirements of signal stability and wear comfort in practical clinical monitoring [53].

At the pattern recognition level, machine learning algorithms were employed to achieve intelligent classification of respiratory behavior. As illustrated in Figure 4j, the decision tree-based respiratory pattern recognition process extracts 12 key features from the acquired respiratory signals for classification. These features encompass time-domain, time-frequency-domain, and frequency-domain information, specifically including: time-domain mean, variance, standard deviation, root mean square, and kurtosis; time-frequency-domain pulse factor, waveform factor, peak factor, and skewness based on wavelet transform; and frequency-domain unbiased estimation, coefficient of variation, and edge factor based on Fourier transform. A classification model was constructed using the training dataset (1580 samples total, with 316 samples per respiratory behavior category) and evaluated on the test dataset (395 data points). This mask-integrated T-TENG system achieved an average recognition accuracy of 97.2% for five typical respiratory behaviors (normal breathing, deep breathing, coughing, sneezing, and laughing). Although the study did not specifically test for sleep apnea, its high-precision classification capability demonstrates potential for distinguishing abnormal breathing patterns (e.g., apnea, nasal/oral/mixed breathing, breath-holding), validating the system’s functional upgrade from “signal acquisition” to “breathing pattern recognition.” This validates the important role of machine learning algorithms in enhancing the intelligence of T-TENG respiratory monitoring. Combined with deep learning models such as convolutional neural networks (CNNs), future work could further enable automatic extraction of respiratory pathological features and early warning, promoting T-TENGs from “monitoring” to “assisted diagnosis.” This lays the foundation for subsequent pathological feature extraction and analysis [118]. Long-term stability represents another critical aspect for engineering applications. As shown in Figure 4k, the long-term stability of WF-TENG was tested in the literature by simulating periodic contact-separation motion using a linear motor at 0.6 Hz frequency and 3.5 kPa pressure. After approximately 5 h of continuous operation, its output voltage decreased from 207 V to 152 V, representing a decay of about 26.6%, demonstrating good output retention capability. These results indicate that WF-TENG exhibits good operational stability in static mechanical testing, providing preliminary support for its long-term use in wearable applications. However, to comprehensively evaluate its engineering potential under actual wear and washing conditions, further environmental simulation and standardized washing tests are required [119].

As shown in Figure 4e, the respiratory signal comparison of the dual IMU differential structure reveals that the peak noise of the unprocessed signal reaches approximately 0.8 V. After differential processing and 0.6 Hz low-pass filtering, the noise is significantly reduced to about 0.1 V, intuitively demonstrating the structure’s effectiveness in suppressing motion artifacts. This study validated the motion artifact resistance across various daily activity scenarios, including sitting, standing, and walking at 1 m/s. Results confirm the system’s ability to reliably extract respiratory signals even during walking, indicating robust performance against motion interference from routine movements [120].

At this point, the discussion of respiratory monitoring as an independent sensing function concludes. The next section will explore the integration schemes and closed-loop applications of T-TENGs in clinical systems.

#### 3.3.4. Preliminary Exploration of Clinical Applicability

In clinical settings, respiratory monitoring technologies can be categorized into wearable and environment-integrated types. As a representative wearable technology, TENG fills the gap for intimate, continuous, and dynamic monitoring, making it particularly suitable for daily activities and long-term wear.

**Figure 5:**
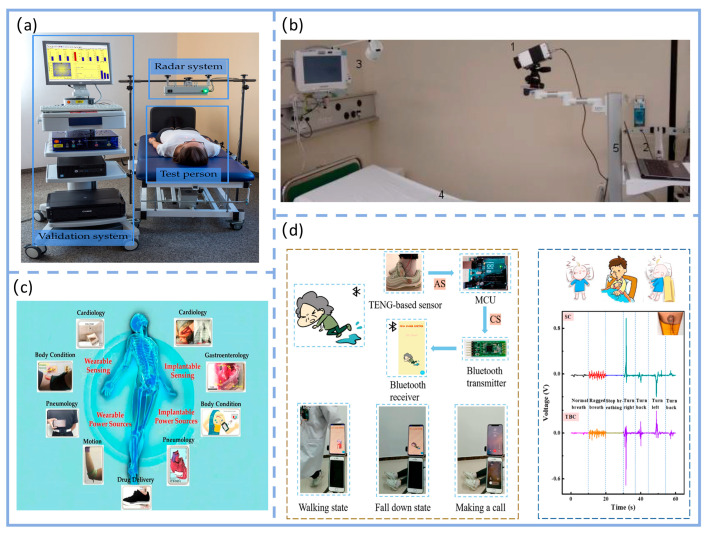
Prototype device, integrated solution, and practical application demonstration of the respiratory monitoring system in clinical settings. (**a**) Photographs of the prototype and validated system. Photographs of the research apparatus. Adapted from Ref. [125]. (**b**) Experimental setup in the Post-Anesthesia Care Unit (PACU) (1) Long-wave infrared camera, VarioCAM^®^ HD Head 820 S/30 mm (Infratec GmbH, Dresden, Germany) (2) Laptop computer (3) Philips IntelliVue MP30 monitor (Philips Electronics N.V., Amsterdam, The Netherlands) (4) Hospital bed (5) Rolling stand. Adapted from Ref. [127]. (**c**) Clinical system integration diagram for smart wearable sensors. Adapted from Ref. [131]. (**d**) Demonstration of SWS for: motion status monitoring and fall alarm system, as well as sleep status monitoring. Adapted from Ref. [85].

As shown in Figure 5a, the interferometric continuous-wave radar system employed in clinical settings demonstrates typical applications for static, non-contact respiratory monitoring. This system was validated in supine position tests involving 30 healthy volunteers (aged 21–61 years, BMI 18.6–31.4 kg/m^2^) in a supine position. This scenario highlights the radar system’s suitability for fixed monitoring environments requiring no physical contact. Meanwhile, wearable technologies such as T-TENG fill the gap in close-fitting, continuous, and dynamic monitoring, making them more suitable for daily activities and long-term wear [125]. Wearable systems require users to carry sensors, which may cause some intrusion but are suitable for mobile scenarios; environmental systems deploy sensors around the user, enabling truly contactless monitoring, particularly suited for clinical static settings. This classification clearly positions T-TENG as an environmental sensor. Its non-contact, wear-free characteristics offer significant advantages in reducing patient discomfort and preventing cross-infection, providing theoretical support for T-TENG’s clinical applicability [121]. The non-contact advantage of environmental systems is further validated in porous silicon capacitive sensors.

Xia et al. developed PDMS sponge-based capacitive sensors integrated into pillows or mattresses, enabling non-intrusive monitoring of supine subjects’ respiratory states. During deep breathing, the relative capacitance change rate reached ~6%, maintaining stable output even after 10,000 cycles of testing [126]. Similarly, as illustrated in Figure 5b, Hochhausen et al. employed long-wave infrared thermal imaging (IRT) technology in a clinical observational study within the post-anesthesia care unit (PACU) to achieve non-contact respiratory rate monitoring of postoperative patients [127]. This technology captures temperature fluctuations around the nasal cavity during inhalation (intake of cool air) and exhalation (expulsion of warm air). By combining particle filter tracking with Bayesian fusion algorithms to extract respiratory waveforms, it demonstrated good correlation with the reference standard of surface electrocardiography (ECG) in respiratory rate detection among 28 postoperative patients (r = 0.607 upon admission, at discharge r = 0.849). It maintained reliable performance even in clinically complex scenarios such as patients requiring nasal cannula oxygen therapy or exhibiting irregular breathing patterns. This provides an additional effective technical pathway for non-invasive respiratory monitoring of sensitive populations (e.g., postoperative recovery patients) in static clinical environments. Compared with static, non-contact monitoring technologies such as infrared thermography, T-TENGs demonstrate significant advantages in dynamic, wearable, and long-term continuous monitoring, particularly in terms of signal real-time capability, wear adaptability, and multi-scenario compatibility. These two approaches complement each other in the field of respiratory monitoring, jointly expanding the application boundaries of non-invasive respiratory monitoring.

TENG exhibits distinct differences from capacitive and piezoelectric sensors in multiple performance metrics, particularly excelling in self-powered capability, flexibility, and cost-effectiveness. As documented in the literature, TENGs leverage triboelectric charging and electrostatic induction principles to directly convert environmental mechanical energy into electrical energy, exhibiting remarkable self-powered characteristics suitable for wireless sensing and wearable devices. Regarding flexibility, polyimide (PI)-based TENGs achieve excellent stretchability and bendability through structures like electrospun fibers and porous aerogels, adapting to complex surface conforming. Cost-wise, TENGs feature relatively simple structures requiring no complex external power supplies or high-frequency signal processing circuits. They can be fabricated using low-cost processes like solution processing and laser-induced graphene (LIG). These characteristics collectively underpin TENGs’ technological competitiveness in flexible sensing applications [128].

Ma, Z et al.’s review illustrates the operating principles and integration schematics of various TENG-based biophysical sensors for respiratory monitoring. For instance, the Respiratory Sensing TENG (RS-TENG) integrated into smart masks enables real-time detection of respiratory rate and breathing status, with circuit modules triggering breathing interruption alerts. Respiratory monitoring TENGs (RM-TENGs) based on nanofiber films generate electrical signals through airflow-driven contact separation, thereby reflecting respiratory timing parameters (inspiration time, expiration time, respiratory ratio, etc.). While these diagrams do not directly illustrate the specific architecture for extracting pathological respiratory features using machine learning or deep learning models, the paper mentions that in similar human motion recognition studies, convolutional neural networks (CNNs) have been employed to analyze multi-channel TENG signals, achieving high-precision classification of diverse neck movements. This indicates that by incorporating temporal signal analysis and pattern recognition algorithms, the respiratory-related electrical signals captured by TENG can be further leveraged to extract pathological features(e.g., respiratory rhythm abnormalities, airflow pattern changes), thereby advancing TENG from mere physiological “monitoring” to “aided diagnosis” with feature analysis capabilities [89].

The textile-based T-TENG has demonstrated core advantages in respiratory monitoring, including self-powering, multi-parameter capability, and high adaptability. However, to achieve the leap from laboratory prototype to clinical implementation, it must confront three core challenges: low-intensity signal noise reduction, cross-scenario stability, and clinical standardization [125]. The T-TENG can establish a correspondence with respiratory waveforms, frequency, and rhythm characteristics output by standard devices such as spirometers by quantifying respiratory timing parameters. Consequently, it provides a viable signal reference foundation for long-term respiratory pattern tracking in COPD patients and automated preliminary screening of sleep-related breathing events, facilitating early identification and dynamic assessment of respiratory disorders in convenient scenarios without traditional polysomnography (PSG) [89]; To realize the transition from laboratory prototypes to clinically applicable systems, parametric optimization methods (e.g., synergistic co-regulation of material, structure, and environment) are of great significance in improving the reliability, repeatability, and engineering standardization of T-TENGs, in line with the methodology required for robust clinical device development. This approach is supported by studies on medical device optimization, such as the application of the Taguchi method for pulmonary ventilators, which demonstrates how systematic parameter tuning can reduce variability and enhance operational robustness—a crucial lesson for T-TENG engineering [129]. Future work needs to further combine multiphysics modeling and clinical validation to promote the standardization and certification process of T-TENGs in respiratory monitoring. Simultaneously leveraging advancements in quantifying triboelectric materials (combining theoretical models with experiments to quantitatively analyze and predict triboelectric charge generation, distribution, and limits) [130] and integration experience with smart wearables (designing and implementing complete wearable systems for real-time health monitoring by integrating TENGs as self-powered sensing units with microcontrollers, wireless communication modules, and terminal analysis software) [85], advancing signal acquisition standardization and system compatibility to bridge the gap from “laboratory excellence” to “clinical significance” [131].

### 3.4. Clinical System Integration and Closed-Loop Applications

As illustrated in Figure 5c, the clinical system integration scheme for smart wearable sensors supports the transition of T-TENG from laboratory prototypes to clinical practicality, enabling a closed-loop system of data acquisition—Transmission—Analysis. During data acquisition, studies confirm TENG sensors can capture key physiological signals with high precision. For instance, woven-structure pressure sensors for cardiovascular assessment achieve sensitivity up to 45.7 mV/Pa, with blood pressure monitoring results showing only 0.87–3.65% deviation from commercial devices. TENG integrated into masks or chest straps reliably extracts respiratory waveforms for identifying sleep apnea events. At the data transmission and system integration level, TENG itself functions as a sustainable micro-energy source, powering complete sensing nodes that include signal processing and wireless transmission (e.g., Bluetooth) to enable real-time collection and remote transmission of data like heart rate. On the data analysis and clinical application side, these signals can not only calculate pulse wave velocity and assess arterial stiffness but also integrate with therapeutic devices to form closed-loop systems. For instance, implantable TENGs have demonstrated the ability to harness heartbeat energy for pacing and arrhythmia correction in animal models, truly realizing the practical clinical circuit of “monitoring-powering-intervention.” Thus, from high-fidelity data acquisition and self-powered wireless transmission to closed-loop analysis directly serving diagnosis and treatment, existing research provides a solid technical foundation and feasibility validation for constructing clinically oriented integrated monitoring systems based on TENGs [131].

As illustrated in Figure 5d, the clinical system integration scheme for smart wearable sensors supports the transition of T-TENG from laboratory prototypes to clinical applications, achieving a closed-loop data acquisition-transmission-analysis system [85]; The comparison of high-humidity (90% RH) output before and after fluorosilane coating treatment shows a voltage retention rate improvement from 60% to 85%,validating the effectiveness of environmental adaptability optimization. Based on the discussion of T-TENG coating processes in this paper, it can be inferred that micro-uniformity and moderate thickness of the coating are critical for maintaining the inherent flexibility of textiles—excessively thick or uneven coatings may infiltrate fabric pores, reducing breathability and deformability, thereby compromising wear comfort and mechanical durability. Furthermore, multiple T-TENG examples in this paper demonstrate that a well-designed coating can significantly enhance output stability without sacrificing flexibility, indicating that the coating process plays a crucial role in balancing environmental adaptability and wear performance [53].

The path from laboratory demonstration to applied respiratory monitoring system is uneven across the different T-TENG approaches. Simple humidity or motion-sensing patches (e.g., [89,99]) often serve as fundamental proof-of-concept for signal detection, lacking the calibrated output and rigorous validation needed for clinical use. Conversely, research on hybrid or multifunctional systems (e.g., [95] combining energy harvesting with wireless sensing, or [91] integrating sensing with air filtration) points toward more integrated application-oriented designs. However, even these advanced concepts typically require extensive field testing, reliability studies under diverse environmental conditions, and regulatory approval processes before they can be deemed ready for practical deployment.

T-TENGs for respiratory monitoring must contend with unique hurdles. The coupling of desired respiratory signals with motion artifacts from body movement is a predominant issue, necessitating advanced sensor designs (e.g., differential structures) or algorithmic filtering that may add complexity. Humidity from breath and perspiration can dramatically alter surface charge properties of triboelectric materials, leading to signal drift or attenuation—a fundamental material-level constraint. Structurally, achieving consistent and comfortable skin-contact without restricting natural chest/abdominal expansion is challenging, especially for integrated garments. Furthermore, the calibration and quantitative translation of T-TENG signal amplitude to absolute volumetric airflow or respiratory effort are not straightforward, limiting their current role to qualitative or relative trend monitoring rather than clinical-grade spirometry. Future research should target solutions that directly address these hurdles: developing sensor arrays with differential layouts or hybrid sensing mechanisms (e.g., combining TENG with inertial or capacitive sensing) to decouple respiratory signals from body motion; employing hydrophobic or moisture-resistant nanocomposite coatings to stabilize charge transfer in humid environments; designing structurally adaptive textiles (e.g., using elastic yarns or auxetic weaves) that maintain consistent contact without restricting movement; and advancing calibration models through machine learning that correlate TENG output with standard respiratory parameters, potentially aided by simultaneous multi-modal data collection.

As shown in Table 5, In the context of respiratory and clinical applications, T-TENG represent an emerging class of self-powered sensing devices with distinct advantages in long-term, continuous monitoring scenarios. Compared to complex biomedical systems optimized through parametric methodologies such as the Taguchi method—as demonstrated in ventilator optimization studies—T-TENG devices currently exhibit lower levels of engineering standardization and reliability under stringent clinical protocols [129]. However, their strengths lie in wearability, low power consumption, self-powering capability, and suitability for unobtrusive, long-term physiological monitoring, which can facilitate early warning and personalized patient management. Future efforts should focus on enhancing the long-term stability of T-TENG devices, integrating AI-driven signal processing algorithms for improved accuracy, and conducting rigorous comparative studies with clinically validated systems. Such steps will support the translation of T-TENG technologies from prototypes to robust, clinically deployable devices, aligning with the parametric optimization paradigm highlighted in recent research.

**Table 5:** Performance overview of selected T-TENGs for respiratory monitoring.

Representative Type	Positive Tribo-Material	Negative Tribo-Material	Electrical Output Performance	Stability (Washability/Durability)	Ref.
Transparent E-Skin TENG	Silver Nanowire Film (Electrode)	Thermally Annealed Poly(vinylidene fluoride) (PVDF) Fibrous Membrane	Voc: 301 V, Isc: 2.7 μA (8N Force), Power Density: 306 mW/m^2^	Exhibits good operational stability and breathability	[132]
Multi-scale Nanofiber Filter TENG	PA66/HACC multi-scale nanofiber membrane	PVDF-HFP nanofiber membrane	Surface potential up to 6.14 kV	Stable under 90% humidity	[133]
Cellulose-based Humidity-Sensitive TENG	Ti_3_C_2_T_x_-modified cellulose template material	Dielectric material (unspecified)	Humidity Sensitivity: 0.8/%RH	Suitable for high humidity (40–90% RH)	[134]
Fabric-based Piezo/Triboelectric Hybrid Sensor	Fabric electrode (e.g., nylon)	ZnO nanorods/polymer composite	Bending sensitivity: 2.59 μA mm	Good stability for long-term wear	[135]

## 4. Motion Monitoring

Human motion monitoring, as a critical technology for assessing physiological status, guiding rehabilitation training, and optimizing athletic performance, has evolved from laboratory-dependent optical systems toward daily health-management tools based on wearable sensors [136,137]. Monitoring solutions represented by inertial measurement units, surface electromyography, and flexible strain sensors have enabled convenient capture of gait, joint movement, and muscle activation patterns [138,139,140]. However, the development of this field remains constrained by several fundamental bottlenecks: most existing sensors rely on external power supplies, and the need for frequent recharging limits the feasibility of long-term continuous monitoring; the mechanical mismatch between conventional rigid or semi-rigid electronic components and human skin or textiles often leads to wearing discomfort, signal drift, or motion artifacts; insufficient environmental robustness of devices makes them susceptible to performance degradation or failure under sweat, temperature-humidity variations, and complex deformations; moreover, many systems are functionally singular, struggling to integrate high-fidelity mechanical sensing, multimodal physiological signal acquisition, and comfortable textile-level wearability on a single platform. Against this backdrop, T-TENGs offer a highly promising pathway to overcome these limitations by virtue of their intrinsic working mechanism that directly converts biomechanical energy into electrical signals. TENGs can serve as self-powered sensors for real-time monitoring of dynamic information such as joint angles, gait phases, and respiratory rhythms, while their material and structural design enables natural integration with textiles. This progress is driving the development of next-generation flexible, autonomous, and sustainable motion-monitoring systems toward greater comfort, intelligence, and reliability [141,142]. The discussion progresses from fundamental motion capture to complex pattern recognition and finally to specialized environments and integrated systems.

### 4.1. Joint Motion and Biomechanical Monitoring

T-TENGs demonstrate significant utility in monitoring fundamental biomechanical activities, particularly the motion of individual joints and the interaction forces involved in daily tasks and rehabilitation. Their flexibility and sensitivity to strain and pressure make them ideal for integration into wearables that track kinematics and kinetics.

**Figure 6:**
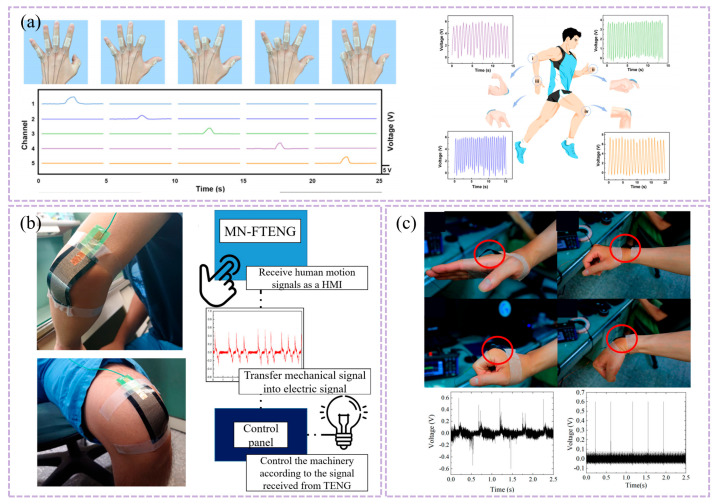
(**a**) The application of the TE-skin regarding human motion monitoring. Color lines are the corresponding voltage signals of the five TE-skins that are fixed on 5 digits. (i) elbow, (ii) finger, (iii) wrist, and (iv) leg. Adapted from Ref. [143]. (**b**) The electric signal of human body motion captured by MN-FTENG. Adapted from Ref. [144]. (**c**) A single-electrode mode TENG is formed by combining the 3% wtGP@PET tribo-layer with human skin, and used to capture joint bending motions and convert them into voltage signals. Adapted from Ref. [145].

This work presents an ultrathin, stretchable all-fiber electronic skin (TE-skin) based on a TENG for self-powered human motion monitoring [143]. The device is fabricated through a scalable electrospinning and spraying process, consisting of a sandwich structure: two thermoplastic polyurethane (TPU) fibrous layers encapsulating a sprayed silver nanowire (Ag NW) electrode. This all-fiber design ensures remarkable mechanical compliance, with the TE-skin capable of withstanding strains up to 500% while maintaining a low electrode resistance of 257.3 Ω at 150% strain. The TE-skin exhibits a high pressure sensitivity of 0.1539 kPa^−1^ and stable electrical output (e.g., ~108 V open-circuit voltage) across a frequency range of 1–5 Hz, which covers typical human motion frequencies. As shown in Figure 6a, its rapid response and excellent signal stability enable real-time, multi-point gesture recognition when integrated into an electronic glove, accurately distinguishing gestures such as “ok,” “victory,” and “good” signs. Furthermore, the TE-skin demonstrates reliable operation under mechanical deformation and varying humidity, underscoring its robustness for wearable sports monitoring. With its superior stretchability, sensitivity, and durability, this TE-skin provides a promising platform for continuous, self-powered motion tracking in smart healthcare and human–machine interfaces.

This work introduces a fiber-based triboelectric nanogenerator (F-TENG) designed for mechanical energy harvesting and human–machine interface (HMI) applications in motion monitoring [144]. The device is constructed using a commercially available polyester fiber coated with a micro-needle structured polydimethylsiloxane (MN-PDMS) layer, paired with an electroless nickel-plated cotton cloth as the flexible electrode. The MN-PDMS microstructure, fabricated via a laser-engraved mold, significantly increases the effective contact area and enhances triboelectric charge generation. Compared to a flat-structured counterpart, the MN-FTENG exhibits a 34% increase in open-circuit voltage (up to 73.6 V) and a 37% increase in short-circuit current (up to 36 µA), with a maximum output power of 1.296 mW. The sensor demonstrates high force sensitivity of 7 V N^−1^ and maintains stable performance over 5000 cycles, along with washability without significant output degradation. As shown in Figure 6b, when attached to human joints (e.g., elbow and knee), the MN-FTENG can capture motion-induced signals with voltage outputs of 0.6 V and 0.8 V, respectively, corresponding to subtle joint forces as low as 0.05–0.1 N. The system integrates with an Arduino-based HMI to enable self-powered control of external devices, showcasing its potential for real-time, low-power motion sensing in wearable health and interactive systems.

Huang et al. recently developed a graphite-doped polyester F-TENG (GP@PET-TENG) by incorporating micron-sized graphite particles into PDMS and coating it onto polyester fabric, resulting in a triboelectric layer that combines high electrical output with wearing comfort [145]. The device employs a vertical contact-separation structure and operates in single-electrode mode. Graphite doping significantly increases the triboelectric charge density and dielectric constant, achieving an open-circuit voltage of 202.1 V and a short-circuit current of 105.1 µA at 3 wt% doping ratio, representing enhancements of approximately 96% and 73%, respectively, compared to the undoped device. As shown in Figure 6c, for motion sensing, when attached to the third knuckle or wrist, the GP@PET-TENG detects fist-clenching and wrist-bending movements in real time, generating voltage signals with peaks around 0.6 V, demonstrating high sensitivity to subtle joint motions. The output synchronizes with motion cycles and remains stable under 7 Hz mechanical excitation, indicating reliable real-time response capability. Although the study does not quantify the linear relationship between motion amplitude and voltage, the consistent correspondence between signals and motions suggests its suitability for qualitative motion monitoring and pattern recognition, providing a robust sensing platform for rehabilitation training and human–machine interfaces.

Beyond isolated joint monitoring, the analysis of coordinated limb movements and whole-body locomotion patterns is crucial for comprehensive motion assessment. This leads to the application of T-TENGs in gait analysis and complex motion pattern recognition.

### 4.2. Gait Analysis and Motion Pattern Recognition

In this domain, T-TENGs are employed to capture dynamic, multi-point pressure distributions and temporal sequences associated with walking, running, and other ambulatory activities. The integration of sensor arrays and advanced data processing algorithms enables detailed locomotion analysis and high-accuracy activity classification.

**Figure 7:**
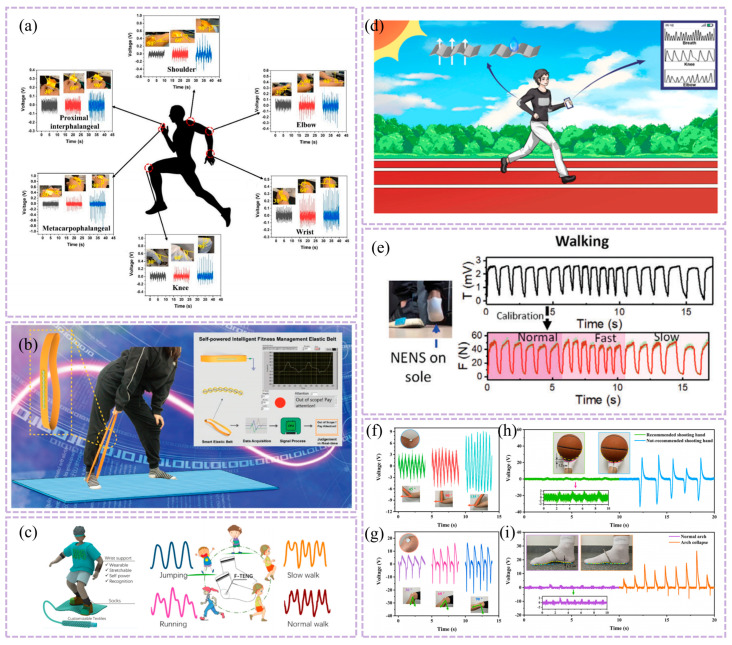
(**a**) T-TENG as a pressure sensor to track physiological movement of an athlete by monitoring the bending angles of different body joints. Adapted with permission from Ref. [146]. Copyright 2025 Elsevier. (**b**) Illustration of the smart fitness system with self-powered yoga belt and real-time monitoring display. Adapted with permission from Ref. [65]. Copyright 2022 John Wiley and Sons. (**c**) Schematic illustration of the F-TENG sock deployed on the foot for various motion. Adapted with permission from Ref. [147]. Copyright 2025 American Chemical Society. (**d**) ISK-TENG Monitoring and Recognition of Diverse Movement Postures. Adapted with permission from Ref. [148]. Copyright 2025 American Chemical Society. (**e**) Continuous human motion monitoring. Adapted with permission from Ref. [149]. Copyright 2024 John Wiley and Sons. (**f**) Detection of arm flexion by attaching the t-TENG to the elbow. (**g**) Detection of the leg swing angles by attaching the t-TENG to the knee. (**h**) Basketball shooting hand monitoring by integrating the t-TENG into the palm position of a glove. (**i**) Foot arch posture monitoring by attaching the t-TENG to the arch position of the sock. Adapted with permission from Ref. [50]. Copyright 2025 American Chemical Society.

Bairagi et al. reported a multifunctional T-TENG based on polyethyleneimine (PEI)-treated cotton fabric. The cotton fibers were modified via a simple and scalable pad-dry process, which significantly enhanced their tribo-positive behavior and imparted antibacterial (83.33%) and antioxidant (74.2%) properties [146]. The device employs a vertical contact-separation structure, with PEI-treated cotton as the tribo-positive layer and a PTFE film as the tribo-negative layer. At a 10% PEI concentration, the T-TENG achieved an output voltage of ~103 V and a current of ~11 μA, representing approximately 3.4-fold and 3.27-fold improvements over the untreated cotton-based device, respectively, and delivered a high power density of ~1600 mW/m^2^. As a motion sensor, the T-TENG can be attached to various body joints (e.g., wrist, elbow, shoulder, knee) to capture joint flexion signals in real time by monitoring contact pressure changes (Figure 7a). Experiments showed that the output voltage increased linearly from 0.2 V to 0.6 V as the wrist bending angle rose from 30° to 90°, demonstrating good angular resolution sensitivity (approximately 4.04 V/kPa). The output signals synchronized with joint motion cycles and remained stable during continuous bending-release tests, indicating reliable real-time monitoring capability. Although the study did not quantify absolute angular error, the strong correlation between signal amplitude and angle variation (R^2^ ≈ 0.99) suggests its suitability for qualitative recognition of joint motion amplitude and patterns, offering a highly adaptable and multifunctional wearable sensing platform for athlete performance analysis and rehabilitation monitoring.

Wu et al. proposed a three-dimensional braided stretchable hierarchical interlocked fancy-yarn TENG (3D HIFY-TENG) with a DNA-like double-wing spiral structure [65]. The yarn employs polyurethane (PU) as the torso yarn, silver-coated polyamide (PA) as the conductive core, and polyimide (PI), polyester (PET), or polyamide (PA) as the insulating sheath, constructing a unique hierarchically interlocked structure via an industrial-scale braiding process. This design enables self-driven contact-separation between the wing yarns and the torso yarn during stretching and recovery, generating triboelectric signals without relying on external objects. The device exhibits excellent mechanical properties (breaking elongation > 350%), weavability, and washability. As a motion sensor, the 3D HIFY-TENG or its fabric can be directly integrated into smart yoga belts or sportswear to monitor joint bending movements (Figure 7b). Research indicates that the electrical output increases significantly when the tensile strain exceeds a critical contact point of approximately 120%, demonstrating good nonlinear strain-response sensitivity. The device output remains stable within the common human motion frequency range of 1–5 Hz, confirming reliable real-time response characteristics. When attached to joints such as the knee, the output signal amplitude positively correlates with the joint bending angle. Although absolute angular calibration was not performed, the consistency between the signal and movement patterns highlights its potential for motion posture recognition and over-limit alarm applications, providing an innovative device architecture and system-level solution for constructing self-powered smart fitness systems.

Zu et al. reported a scalable and customizable T-TENG based on a stretchable coaxial fiber structure [147]. The device was fabricated via a two-step braiding process, featuring a polyurethane (PU) fiber as the elastic core, a braided silver layer for conductivity, and an outer polyamide (PA) layer as the triboelectric material, forming a PU/Ag/PA coaxial structure with a diameter of about 1 mm. This design offers excellent stretchability (strain > 60%) and a stable conductive network. In terms of sensing performance, the F-TENG exhibits a sensitivity of 0.554 V·kPa^−1^ in the low-pressure region (<30 kPa), a wide working range of 5–150 kPa, and response/recovery times of approximately 205 ms and 229 ms, respectively, demonstrating rapid pressure responsiveness. By integrating the F-TENG into customizable textiles such as smart socks and wristbands, the device can capture real-time motion signals from the foot and wrist joints. As shown in Figure 7c, coupled with a one-dimensional convolutional neural network (1D-CNN) algorithm, the system achieves a recognition accuracy of up to 99% for five human motion patterns (standing, slow walking, normal walking, running, and jumping), highlighting its strong potential for motion pattern classification and identity recognition. This work provides a feasible technical pathway for the large-scale production of high-performance, wearable, and self-powered textile motion sensors.

Yin et al. reported a Janus F-TENG with an interlock stitch knitted structure (ISK-TENG) for multimodal physiological signal monitoring such as motion and respiration [148]. The device employs cotton/silver-plated PA6 core-spun yarn and PTFE monofilament as positive and negative triboelectric materials, forming a parallel tubular Janus structure through interlock knitting, which exhibits excellent stretchability (withstanding 50% strain), breathability, and durability (stable performance after 10,000 cycles). Under lateral stretching, the ISK-TENG delivers an output of 160 V and 130 nA, while under vertical pressing it reaches 6 V and 15 nA, demonstrating dual-mode responsiveness to both tensile and compressive deformations. The response time is approximately 190 ms (activation) and 150 ms (recovery) in stretching mode, and about 120 ms (activation) and 210 ms (recovery) in pressing mode, indicating promising real-time monitoring capability. Integrated into the knee area of sports pants, the device can simultaneously capture signals from joint movements and respiration-induced fabric deformation. Using a recurrent neural network (RNN) for signal analysis, it achieved 100% recognition accuracy for different breathing patterns (fast, medium, slow) and 96.8% accuracy for joint motion identification (Figure 7d). This work, through material selection and structural innovation, provides a new approach to addressing the limitations of conventional wearable sensors in comfort, multimodal sensing, and signal accuracy.

Dong et al. reported an innovative wearable nano-energy-nano-system (NENS) by integrating a T-TENG with a high-performance aluminum nitride (AlN) photonic modulator for continuous and self-sustainable force sensing and motion monitoring [149]. The system features a hybrid flexible-rigid architecture: the T-TENG module employs conductive textiles paired with Ecoflex (negative) and nitrile (positive) tribo-layers, forming a breathable, wearable structure suitable for integration into garments; the AlN photonic module is a precisely fabricated micro-ring resonator (MRR) on a silicon substrate, leveraging the strong Pockels effect of AlN. This integration creates a synergistic effect where the T-TENG’s high-voltage output (over 300 V) directly drives the voltage-sensitive AlN modulator, bypassing the need for external power. The system exhibits a wide and tunable force-sensing range up to approximately 200 N, with the optical response showing a monotonic and speed-independent relationship to the applied force magnitude, a significant advantage over conventional speed-sensitive TENG readouts. The response time of the photonic sensing link is fundamentally limited by the photon lifetime in the high-Q MRR (~40 ps), enabling real-time tracking of dynamic forces. Through a derived physical model that correlates the optical transmission shift with the applied force, the system achieves accurate and quantitative force readout. As shown in Figure 7e, in practical demonstrations, the NENS successfully monitored continuous human motions such as finger tapping and walking gaits, translating optical signals into real-time force profiles with high fidelity. This work, by merging triboelectric energy harvesting with integrated silicon photonics, provides a novel paradigm for building self-powered, high-accuracy, and continuous motion monitoring systems for next-generation smart healthcare.

Zheng et al. developed a single-electrode r based on an all-textile structure (t-TENG) [50]. The device employs a conductive fabric coated with a PVDF-HFP/BaTiO_3_ nanocomposite as the negative tribolayer, paired with a highly positive glass-fiber fabric, and is flexibly assembled using knotted yarns, resulting in a breathable, tailorable, foldable, and lightweight fully textile integrated system. Through synergistic optimization of dielectric modulation and electrical poling, the t-TENG (2 × 2 cm^2^) delivers a high output of 261 V, 1.5 μA, and 12.7 nC, with an instantaneous power density of 654.48 mW·m^−2^, and maintains stable performance over 20,000 cycles. As a self-powered pressure sensor, it exhibits a sensitivity of 3.438 V·kPa^−1^ in the low-force region (<10 N) with excellent linearity (R^2^ > 0.97). In practical motion monitoring, the t-TENG can be attached to joints to detect bending angles of the arm and knee, with output signals increasing correspondingly (e.g., 3.2–8.9 V for arm flexion from 45° to 135°) (Figure 7f,g). Notably, by integrating the device into a glove and a sock, it effectively distinguishes basketball shooting-hand types (recommended hand: ~2 V signal; non-recommended hand: ~30 V signal) and foot-arch postures (normal arch: <2 V signal; arch collapse: 10–20 V signal), demonstrating high discriminative capability for sport-specific postures (Figure 7h,i). Although the response time was not explicitly quantified, its fast-response characteristic is validated in real-time motion monitoring. This work, through synergistic material-structure-performance design, provides an important example of highly comfortable and sensitive T-TENGs for intelligent sports posture monitoring and correction.

The practical deployment of T-TENG-based motion monitoring systems often demands robustness against harsh environmental conditions and seamless integration with power management and communication modules. The following subsection highlights advances addressing these challenges.

### 4.3. Applications in Extreme Environments and System Integration

Extending the use of T-TENGs to demanding scenarios such as high-temperature workplaces or humid conditions requires material and structural innovations. Furthermore, the transition from functional devices to practical solutions hinges on effective system-level integration for energy autonomy and data handling.

**Figure 8:**
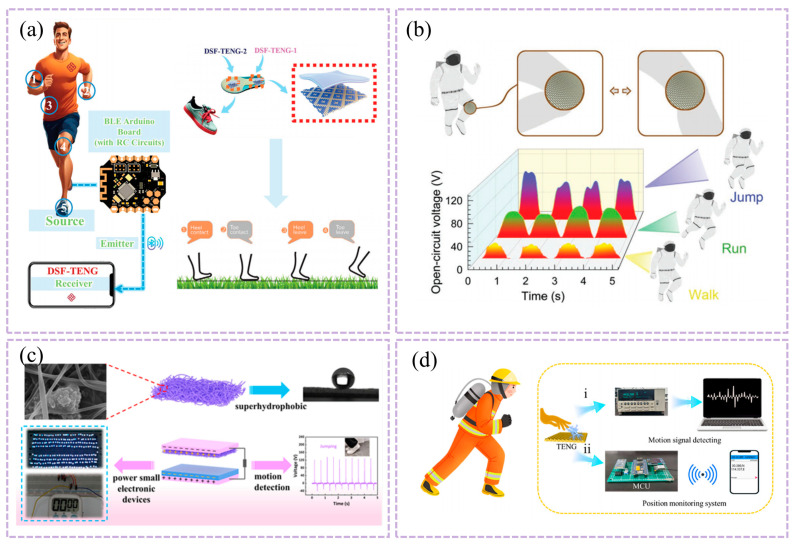
(**a**) Utilizing DSF-TENG for intelligent sensing applications. Adapted with permission from Ref. [150]. Copyright 2024 John Wiley and Sons. (**b**) Fixed location of Y-TENGs and outputs of different motion states. Adapted with permission from Ref. [151]. Copyright 2022 John Wiley and Sons. (**c**) Application of HPP-TENG as a self-powered sensor. Adapted with permission from Ref. [152]. Copyright 2025 American Chemical Society. (**d**) Schematic diagram of the construction of intelligent clothing and a real-time location positioning system. (i) Using an electrometer (6517A) to measure the signal generated by the FPIT-TENG. (ii) The firefighter position monitoring system is composed of a TENG, a microcontroller unit (MCU), a Bluetooth module, and a mobile phone. Adapted with permission from Ref. [153]. Copyright 2025 American Chemical Society.

Ahmed et al. proposed a diamond-structured F-TENG (DSF-TENG) fabricated via a facile, economical, and scalable weaving process without chemical modification [150]. The positive tribolayer consists of a 3D diamond-patterned fabric woven from cotton/silver-plated nylon yarn, while the negative layer is a laminated structure of BaTiO_3_-doped PDMS composite film and copper-nickel conductive fabric. The unique 3D diamond topology significantly increases the effective contact area (≈9.13% larger than a flat surface) and enhances mechanical interlocking, thereby greatly improving charge generation and transfer efficiency. Under conditions of 5 Hz and 15 N, the DSF-TENG delivers an outstanding output of ≈763 V, a short-circuit current of ≈20.4 µA, and a power density of 2862.78 mW·m^−2^, surpassing most reported fabric-based TENGs. The device also exhibits excellent air permeability (560 mm·s^−1^), washability (stable performance after 10 washing cycles), and durability (stable output over 30,000 cycles). As shown in Figure 8a, in motion monitoring applications, the DSF-TENG can be attached to joints to detect bending angles (e.g., 150 V signal for 60° elbow flexion) and has been successfully integrated into an insole for gait analysis, walking-speed monitoring, and real-time detection of abnormal gait and falls in Parkinson’s disease patients. This work, through structural innovation, achieves a balance between high performance and wearing comfort, providing an important example for intelligent motion monitoring and healthcare applications based on T-TENGs.

Xing et al. developed an aerogel nano-covered yarn-based triboelectric nanogenerator (Y-TENG) fabricated via a two-step process combining electrospinning and traditional twisting [151]. The yarn features a core–shell structure, with carbon fiber and PTFE serving as the conductive core and dielectric layer, respectively, while the shell is composed of a polyimide (PI) and silica aerogel nanocomposite. This design provides remarkable thermal stability (withstanding up to 400 °C), flame retardancy, flexibility, and fineness (≈1 mm diameter), making it suitable for weaving into wearable textiles. In terms of motion monitoring performance, the Y-TENG exhibits high sensitivity to various mechanical stimuli such as pressure, strain, and joint movements. It achieves a fast response time of less than 15 ms, delivers an open-circuit voltage of 80 V and a transferred charge of 27 nC under 50 N force, and maintains stable electrical output across a broad temperature range of 25–400 °C. Leveraging its high-temperature resistance and structural robustness, the Y-TENG has been integrated into smart protective clothing to monitor motion signals from multiple body areas including the back, neck, knees, elbows, and shoulders. By analyzing signal amplitude, frequency, and waveform characteristics, the system can accurately distinguish between different motion states such as walking, running, and jumping, offering real-time, self-powered monitoring and rescue support for workers in high-risk environments (Figure 8b). This study demonstrates a material-and-structure co-design strategy that enables reliable motion sensing in extreme conditions, extending the potential of T-TENGs for smart healthcare and motion monitoring applications in high-temperature and hazardous scenarios.

Qu et al. developed a superhydrophobic nanofiber membrane-based T-TENG (HPP-TENG) that combines flexibility, eco-friendliness, and moisture resistance for biomechanical energy harvesting and self-powered motion monitoring [152]. The tribopositive layer (HPP-NF) was fabricated via electrospinning followed by spray coating: using biodegradable polycaprolactone (PCL) as the matrix, doped with conductive polyaniline (PANI) to enhance charge transfer and tribopositivity; subsequently, hexadecyltrimethoxysilane (HDTMS) and silicon dioxide (SiO_2_) were sprayed to lower the surface energy and construct micro-nano roughness, resulting in a superhydrophobic membrane with a contact angle of 154°. This membrane was paired with a PTFE film to form a contact-separation mode HPP-TENG. Through synergistic material and structural optimization, the HPP-TENG delivers an open-circuit voltage of 150 V and a short-circuit current of 11 μA under 10 N at 3 Hz, with a maximum power density of 75.6 μW/cm^2^. Its superhydrophobicity effectively suppresses charge dissipation in humid environments (55% RH), with a rapid recovery time of only 35 s, ensuring signal stability under practical conditions. In motion monitoring applications, owing to its high sensitivity and fast charge response, the HPP-TENG can accurately distinguish and quantify human motions of different intensities and modes: when attached to joints or clothing, it generates real-time voltage signals positively correlated with movement amplitude and force, such as finger tapping (7 V), elbow bending (18 V), arm lifting (58 V), walking (35 V), jumping (120 V), and running (150 V) (Figure 8c). Although the study did not provide systematic metrics for response time or classification accuracy, the clear correlation between output signal amplitude and motion intensity, coupled with stable performance over 3000 cycles, demonstrates its potential as a sustainable and durable self-powered motion sensor. This work provides a material and device foundation for long-term, stable smart healthcare motion monitoring in humid or daily activity environments.

Hao et al. developed a T-TENG (FPIT-TENG) woven from fluorinated polyimide yarns (FPIY) for motion and position monitoring in high-temperature and high-humidity environments [153]. The device features a core–shell yarn fabricated via conjugate electrospinning: a nickel-plated aramid yarn serves as the conductive core, while the shell consists of fluorinated ethylene propylene (FEP)-doped fluorinated polyimide (FPI) nanofibers. The introduction of fluorinated groups significantly enhances the electron affinity and thermal stability of the material, and FEP doping further improves surface charge density and hydrophobicity. Benefiting from the unique micro-nano hierarchical porous structure, the yarn exhibits outstanding electrical output in single-electrode mode (open-circuit voltage of 22.7 V per 10 cm length). The woven fabric demonstrates excellent high-temperature resistance (operable up to 250 °C), superhydrophobicity (contact angle of 147°), and moisture permeability. At 99% relative humidity, the FPIT-TENG retains about 80% of its electrical output and shows no significant degradation after 10,000 cycles, indicating remarkable environmental adaptability and durability. For motion monitoring, the FPIT-TENG was integrated into firefighting clothing. By detecting contact–separation signals generated during body movement, it can distinguish between different motion intensities and modes: slow walking, fast walking, and running correspond to output voltages of approximately 5.2 V, 9.2 V, and 9.5 V, respectively, with signal waveforms (unidirectional/bidirectional) and frequencies (0.7 Hz, 1.9 Hz, 1.5 Hz) reflecting motion amplitude and contact status. Although the study did not provide systematic metrics for response time or classification accuracy, a clear and stable correlation between output signals and motion intensity/patterns was demonstrated. Furthermore, by incorporating a Bluetooth module and GPS, real-time location tracking and motion status feedback for firefighters were achieved (Figure 8d). This work, through material fluorination, hierarchical structuring, and system integration, presents a feasible T-TENG solution for reliable, self-powered motion and position monitoring in extreme environments.

The majority of reported T-TENG motion sensors, particularly those demonstrating single-joint monitoring or basic activity recognition in controlled lab environments (e.g., [119,120,121,122]), are best classified as versatile proof-of-concept platforms. They validate the sensing mechanism but often lack the systematic robustness testing, quantitative calibration, and user-centric design needed for applied use. Notable exceptions moving toward application-specific systems include the DSF-TENG integrated into an insole for gait analysis and fall detection in Parkinson’s patients [127], and the Y-TENG or FPIT-TENG designed for high-temperature environments [128,130]. These works explicitly address performance under real-world stressors (repetitive use, washing, extreme temperatures), marking a transition from conceptual validation to engineered solutions for targeted scenarios.

Extending T-TENGs to dynamic motion monitoring exposes critical limitations. The nonlinear and often hysteretic relationship between electrical output and mechanical input (strain, pressure) complicates the derivation of precise quantitative metrics (e.g., joint angle in degrees, force in Newtons), often limiting use to qualitative movement detection or pattern recognition. Signal cross-talk in densely integrated sensor arrays and degradation of performance under extreme mechanical deformation (e.g., over-stretching, twisting) are recurring structural concerns. From a system perspective, the high-impedance, alternating-current nature of TENG output necessitates tailored power management circuits for efficient energy storage and stable power supply to downstream electronics, adding to integration complexity. Finally, the lack of standardized testing protocols and performance metrics for textile-based TENG sensors hinders direct comparison between studies and slows progress towards established reliability standards. To overcome these limitations, future efforts could focus on: developing hybrid sensor designs that combine TENGs with linear-response sensors and applying machine learning models to map nonlinear outputs to quantitative metrics; optimizing array layouts and incorporating shielding or ground layers to minimize cross-talk; exploring ultra-stretchable conductors and structurally resilient fabric architectures to maintain performance under deformation; integrating custom-designed, high-efficiency power management units (PMUs) directly onto flexible substrates; and initiating community-wide efforts to establish standardized testing protocols for key motion-sensing parameters under realistic wearing conditions.

This section demonstrates that T-TENGs offer a distinct approach to motion monitoring, as detailed across applications in joint and biomechanical monitoring, gait and complex pattern recognition, and specialized environments with system integration. As shown in Table 6, T-TENGs offer a distinct approach to motion monitoring, differing from conventional externally powered and rigid sensors, through their self-powered mechanism and innate morphological compatibility with textiles. Their advantages in wearing comfort, environmental robustness, and multimodal mechanical sensing, combined with machine learning algorithms, have enabled high-accuracy recognition from joint movements to full-body postures. However, challenges remain in transitioning from lab demonstrations to practical applications: establishing standardized, precise quantitative relationships between sensing signals and physiological parameters is urgently needed; the long-term reliability of devices under dynamic and complex real-world conditions requires deeper investigation; ultimately, system integration and rigorous clinical validation are essential to advance them into truly practical tools for smart healthcare and sports science.

**Table 6:** Performance comparison of representative T-TENGs for motion monitoring across different application themes.

Representative Type	Positive Tribo-Material	Negative Tribo-Material	Electrical Output Performance	Stability (Washability/Durability)	Ref.
Core–Shell Structured Nanoyarn TENG	Silver-coated nylon yarn (Conductive core)	PVDF-TrFE/Cs_3_Bi_2_Cl_9_ composite sheath layer	Sensitivity: 3.64 V/kPa; Durability: >50,000 cycles	Wear-resistant and durable. Integrated core–shell structure ensures high mechanical stability.	[106]
Highly Stretchable Coaxial Fiber TENG	Human skin/FEP-Silicone rubber composite outer layer	MWCNT-Silicone rubber composite inner core electrode	Voc~8 V (single fiber tapping); Power density: 1.794 μW/cm^2^; Stretchability: 874%	Washable and highly stretchable.	[154]
Multifunctional Integrated Smart Fabric TENG	Polydopamine (PDA)-modified phase-change fiber layer	PVDF-HFP/Graphene/CNT composite film	Voc~77 V; Power density: 8762 μW/m^2^	Maintains >93% performance after 10,000 compression cycles, 10-month storage, and washing.	[155]
All-Textile Embedded Electrode TENG	Wool yarn	Polyester yarn	Voc~18.5 V; Power density: 51 mW/m^2^	Demonstrates good washability, durability, and all-weather adaptability.	[156]
Electrospun Composite Film TENG	Nylon film	TPU/PVDF electrospun fiber film	Max output power: 699 μW (at 7 MΩ); Pressure sensitivity: 14.08 V N^−1^	Good abrasion resistance, suitable for integration into footwear, etc.	[157]

## 5. Conclusions

### 5.1. Challenges and Prospects of T-TENGs

T-TENGs exhibit immense application potential in monitoring vital signs such as pulse, respiration, and sleep. By detecting subtle skin changes induced by heartbeats to accurately record pulse signals or analyzing periodic electrical signal variations resulting from chest movements to infer respiratory frequency and patterns, T-TENGs demonstrate notable advantages in high precision and real-time performance. However, moving towards the large-scale practical application of human health and sports monitoring, T-TENG still faces a series of severe challenges. The primary bottleneck lies in its limited output performance. Constrained by factors such as contact area, material surface charge density, and the low-frequency mechanical energy input from human motion, its output power typically ranges from microwatts to milliwatts. While this is sufficient to power micro-sensors, it struggles to directly support more complex electronic devices with higher power demands. This can be addressed by employing advanced spinning technologies to produce conductive yarns that are lightweight, highly conductive, flexible, and have low linear density. By integrating these yarns into various traditional textile structures (e.g., full-fashioned knitting, weaving, braiding, non-woven fabrics) tailored for specific application scenarios, it is possible to enhance the electrical output, comfort, wear resistance, sensing precision, and sensing range of T-TENGs, thereby enabling more effective monitoring of human health and motion.

Secondly, issues of reliability and durability are prominent. Long-term and repeated friction can lead to wear, delamination, or microstructural damage in the functional material layers, resulting in performance degradation. Furthermore, unavoidable factors in daily use, such as sweat, laundering, and environmental humidity fluctuations, can significantly interfere with charge generation, transfer, and retention, causing output instability or even failure. These challenges can be mitigated by utilizing core-spun yarn blending techniques to protect conductive components from performance decay, and by employing yarns with high wear resistance and applying appropriate finishing agents to impart additional functionalities, meeting the demands of diverse application scenarios.

Thirdly, the manufacturing and integration processes are complex. To achieve high performance, it often necessitates nano-material coating, doping, or surface modification at the fiber level. The compatibility of these processes with large-scale textile production, along with cost control and yield rate, poses significant challenges. This can be approached by refining ultrafine fiber manufacturing processes to address coating issues at the fiber and yarn stage before proceeding with fabric structure design.

Moreover, medical-grade data demands extremely high accuracy and stability. The output signals of T-TENGs are susceptible to interference from environmental humidity, bodily sweat, and non-uniform fabric deformation. Extracting pure, calibratable physiological parameters (such as precise cardiopulmonary function indicators or joint range of motion) from these complex electrical signals is a critical challenge for both algorithm and sensor structure design. This can be tackled by leveraging AI technologies to continuously refine algorithm accuracy and adjust sensing sensitivity.

Finally, constructing a complete self-powered system is not straightforward. T-TENGs generate high-voltage, low-current, pulsed alternating current. They must be coupled with efficient power management circuits (including rectification, voltage regulation, and energy storage modules, such as micro-supercapacitors) to form a stable power source. This increases system complexity, size, and overall design difficulty.

Therefore, future research must transcend iterative device description and adopt a co-design philosophy targeting these core limitations. Promising directions include: developing novel composite fibers and coatings with intrinsically high and stable triboelectric activity, environmental resistance, and mechanical resilience; innovating hierarchical fabric structures that decouple and optimize sensing, power generation, and comfort functions; designing embedded hybrid systems that integrate T-TENGs with complementary sensing modalities (e.g., capacitive, resistive) and efficient on-textile power management to provide more robust and quantitative data; and establishing universal performance evaluation standards under realistic wearing and washing conditions to enable meaningful benchmarking. By addressing these critical challenges, the transition of T-TENGs from compelling laboratory prototypes to reliable, market-ready technologies for personalized health and performance monitoring can be accelerated.

The burgeoning role of artificial intelligence (AI) and machine learning (ML) in wearable sensing presents a transformative, yet underexplored, frontier for T-TENG development. While the primary research focus to date has rightly been on enhancing fundamental device performance (output, stability, sensitivity) and textile integration, the sophisticated, often non-linear and multi-parametric signals generated by T-TENGs are inherently suited for advanced data-driven interpretation. ML algorithms can play a pivotal role in overcoming critical bottlenecks: de-noising signals contaminated by motion artifacts or environmental interference, extracting subtle physiological features from complex waveforms, classifying diverse activity or health states with high accuracy, and even calibrating sensor output to quantitative physical metrics. However, effective ML integration faces its own challenges, including the need for large, high-quality, and accurately labeled datasets collected under realistic conditions, the development of lightweight models suitable for deployment on edge devices, and the inherent difficulty in interpreting ‘black-box’ models for clinical or safety-critical applications. Future research must therefore foster deeper collaboration between material scientists, textile engineers, and data scientists. A key direction is to move beyond using ML merely for post hoc analysis and towards the co-design of T-TENG systems and algorithms, where sensor architecture and data acquisition are optimized synergistically to generate signals that are both information-rich and algorithmically tractable, ultimately unlocking the full potential of intelligent, self-powered textile sensing systems.

Furthermore, moving beyond empirical optimization, the integration of finite element method (FEM) modeling with artificial intelligence (AI) presents a powerful computational paradigm to accelerate the rational design of T-TENGs. FEM can precisely simulate the distribution of mechanical stresses, contact area evolution, and electrostatic potential fields within complex textile structures under dynamic wear conditions. Coupled with AI algorithms, this enables the exploration of vast design spaces for optimal performance. Such a combined approach can guide the inverse design of yarn architectures, fabric weaves, and composite layers to maximize triboelectric output, ensure mechanical robustness, and predict long-term behavior, thereby reducing reliance on trial-and-error and fostering a more fundamental, predictive science of textile-based energy harvesting and sensing.

In conclusion, T-TENGs represent a promising yet still maturing technology. Future development will focus on the goals of “high performance, high reliability, and mass producibility.” This will be achieved by developing novel composite materials with high charge density, designing multi-level microstructures resistant to fatigue, researching environmental isolation and encapsulation strategies, optimizing manufacturing processes compatible with the textile industry, and promoting the monolithic integration with energy storage units and electronic components. Ultimately, this will enable the transition from laboratory prototypes to market-competitive products, paving the way for broad application prospects in the fields of non-invasive, real-time, and convenient smart healthcare and sports monitoring.

### 5.2. Enhancing Experimental Reproducibility and Standardization

Achieving high experimental reproducibility is a cornerstone for translating T-TENG research into reliable technologies. The perceived variability often stems from several intertwined factors inherent to textile-based systems: (i) Textile Material Inherent Variability: Commercial textiles (e.g., yarn fineness, surface morphology, weave/knit density, fiber blend ratio, and pre-treatment finishes) can vary between batches and suppliers, directly impacting triboelectric properties and device-to-device consistency. (ii) Environmental Sensitivity: The intrinsic hygroscopic nature of many natural and synthetic textile fibers and the charge dissipation in humid environments lead to significant output fluctuation unless strictly controlled. (iii) Textile-Integrated Process Dependency: Key fabrication steps (fiber functionalization, yarn coating, fabric finishing) are sensitive to subtle variations in parameters (concentration, temperature, time, tension, and machine settings), which are often less controlled than in microfabrication. (iv) Lack of Unified Metrics: The absence of standardized testing protocols specific to textile-based devices (e.g., for applied pressure profile on curved fabric surfaces, frequency, electrode configuration, as well as textile-specific metrics like abrasion cycles, washability, and breathability) hinders direct cross-laboratory comparison.

To overcome these challenges, a multi-faceted strategy is essential. First, establishing standardized textile material benchmarks (e.g., specifying fiber type, yarn count, fabric structure, and sourcing) and detailed textile-compatible fabrication protocols for reporting is crucial. Second, adopting controlled environmental chambers for characterization can decouple performance from ambient fluctuations. More proactively, the field should move towards textile-oriented intelligent manufacturing and monitoring. This includes employing machine learning (ML) not just for data analysis, but for optimizing and monitoring textile fabrication and functionalization processes (e.g., using vision systems to ensure fiber coating uniformity across yarn lengths) and for developing on-the-fly signal compensation algorithms that can correct for known environmental drift based on real-time sensor data and textile material properties. Ultimately, community-driven initiatives to establish consensus-based textile-TENG-specific testing standards, potentially drawing from both textile industry norms and electronics testing protocols, will be indispensable for building a robust, reproducible knowledge base and accelerating credible technological progression.

### 5.3. Considerations for Clinical Translation

A critical, yet frequently underexplored, dimension in the roadmap from laboratory innovation to clinical deployment is compliance with medical device regulations and certification standards. The current T-TENG literature predominantly focuses on performance metrics and proof-of-concept demonstrations, with limited explicit discussion of the regulatory landscape. For any T-TENG-based system intended for patient monitoring or therapeutic intervention, adherence to frameworks such as the International Organization for Standardization (ISO) standards for medical devices (e.g., ISO 10993 for biological evaluation [158], ISO 13485 for quality management systems [159]) and regional regulations like the European Union’s Medical Device Regulation (MDR) or the U.S. Food and Drug Administration (FDA) requirements becomes paramount. These frameworks mandate rigorous assessment of biocompatibility (for long-term skin contact), electrical safety, electromagnetic compatibility, mechanical and environmental robustness (including under repeated washing and sterilization cycles), long-term stability, and clinical performance validation. The unique nature of T-TENGs—integrating energy harvesters, sensors, and textiles—poses novel classification and testing challenges. Future research aimed at genuine clinical impact must, therefore, integrate regulatory thinking early in the design phase. This involves selecting materials with established biocompatibility, designing for manufacturability and consistent quality, developing protocols for accelerated life and reliability testing under realistic use conditions, and engaging with clinical and regulatory experts to define appropriate performance benchmarks and risk management strategies. Proactive attention to these requirements is not merely a final hurdle but a necessary foundation for building trustworthy, safe, and effective smart textile-based medical technologies.

### 5.4. Guidelines for Translating Laboratory T-TENGs to Practical Applications

The transition of T-TENGs from compelling laboratory demonstrations to reliable, market-ready technologies necessitates a concerted research focus on overcoming the intertwined challenges of charge retention, interface stability, long-term durability, and scalable manufacturing—all within the context of textile substrates and processes. Future work should pivot towards rational, application-targeted textile material and device engineering, moving beyond incremental improvements to establish clear design guidelines for practical systems.

Rational design and interfacial engineering of next-generation high-performance and environmentally stable textile-based triboelectric materials are key to addressing the core challenges of charge retention and interfacial stability. A pivotal direction involves the rational molecular and structural design of triboelectric materials that are compatible with and can be effectively integrated into textile matrices (fibers, yarns, fabrics) to intrinsically enhance charge density and environmental stability. For instance, the development of high-tribo-polarity polymers, such as sulfur-rich copolymers, through controlled synthesis or functionalization for fiber spinning or fabric coating has demonstrated the potential to generate large electrical outputs even under weak mechanical stimuli, directly addressing the need for high sensitivity in wearable scenarios [160,161,162]. Concurrently, interfacial engineering must focus on developing durable bonding layers, self-healing materials, or covalently grafted functional coatings specifically formulated for fibrous and porous textile surfaces to mitigate delamination and performance degradation in dynamically flexing textiles. The synergy between bulk material modification (e.g., dielectric constant enhancement via textile-compatible nanofillers like MXenes [162]) and surface engineering (e.g., durable hydrophobic/oleophobic treatments for textiles) will be crucial for maintaining stable triboelectric performance in the presence of sweat, humidity, and repeated mechanical stress while preserving textile handle and comfort.

Investigating emerging paradigms such as ultrasound-driven energy harvesting and sensing, combined with scalable textile manufacturing processes and standardized evaluation protocols, will pave the way for the application of T-TENGs in non-contact and implantable systems, as well as in large-scale implementations. Beyond optimizing traditional contact-based modes, exploring novel operational paradigms can unlock new application spaces. Ultrasound-driven T-TENGs represent a transformative avenue for non-contact energy harvesting and sensing, enabling power transfer and physiological monitoring through biological tissues or sealed environments [163,164,165]. This technology is particularly promising for wirelessly charging implantable medical devices, powering on-demand transient electronics, and creating truly unobtrusive sensing platforms. For textile integration, this could inspire the development of non-contact sensing fabrics that monitor vital signs without direct skin pressure. To translate these and other laboratory innovations, future efforts must rigorously address scalability and reliability within textile manufacturing frameworks. This includes developing manufacturing techniques directly compatible with the textile industry (e.g., roll-to-roll processing of functional coatings, high-speed yarn functionalization lines, and digital weaving/knitting of integrated circuits using functional yarns) and establishing standardized protocols for evaluating key parameters under realistic textile wear conditions (e.g., charge decay rate over thousands of flexing and abrasion cycles, performance after standardized industrial washing, and output stability under varying temperature/humidity while maintaining textile properties like breathability). A holistic co-design approach, which simultaneously considers textile-compatible material properties, fabric structure, textile-integrated power management electronics, and user-centric design, will be indispensable for bridging the gap between proof-of-concept prototypes and robust, commercially viable T-TENG products for smart healthcare and sports science.

## Figures and Tables

**Table 1:** Summary of T-TENG architectural hierarchy: material composition, fabrication methods, advantages, and typical electrical outputs.

Architectural Level	Positive Materials	Negative Materials	Typical Fabrication Methods	Advantages	Typical Electrical Output
Fiber	Nylon, Polyamide (PA), Silk, Polyimide (PI)	PTFE, PVDF, FEP, PDMS composites	Electrospinning, Wet-spinning, Melt-spinning, Dip-coating, CVD	High surface-area-to-volume ratio; tunable triboelectric properties via material selection and surface engineering; enables micro-scale charge generation.	Voltage: 10–150 V; Current: 0.1–10 µA; Power density: 0.1–10 mW/m^2^
Yarn	PA, Silk-coated yarns	PTFE-sheathed, PDMS-coated yarns	Twisting, Braiding, Covering/Wrapping, Fancy yarn spinning	Mechanical robustness; strain distributable; scalable length production; maintains flexibility and durability under deformation.	Voltage: 20–200 V; Current: 0.5–20 µA; Power density: 1–50 mW/m^2^
Fabric	Nylon-based woven/knitted fabrics	PTFE/PVDF-coated textiles, PDMS-laminated fabrics	Weaving (Loom), Knitting (Circular/Flat bed), Non-woven processes, Lamination	Wearable, breathable, conformable; programmable macro-scale contact modes; suitable for large-area sensing and energy harvesting.	Voltage: 50–300 V; Current: 1–30 µA; Power density: 10–500 mW/m^2^

**Table 2:** Structural Classification and Performance Comparison of Textile-Based TENGs for Pulse Wave Monitoring.

Structural Category	Representative Structures	Sensitivity	Response Time	Cycling Stability	Pulse Monitoring Capability
Thin-film	Ultra-flexible sensor (UFS)	~0.12–0.25 V Pa^−1^	<20 ms	>50,000 cycles	Radial artery; pulse waveform & HRV
Electrospinning	Sandpaper-molded/TPU nanofiber array	1.67 V kPa^−1^ 0.20 V kPa^−1^	~30 ms	>7200 cycles	Carotid & wrist pulse
All-fiber/Yarn	All-fiber structured pressure sensor	1.33 V kPa^−1^ 0.32 V kPa^−1^	~40 ms	>10,000 cycles	Neck & wrist pulse
Fabric-based	Textile-based sensor array (TATSA)	~1.33 V kPa^−1^	~20–30 ms	>10,000 cycles	Multi-site pulse monitoring

**Table 3:** Structural design strategies and mechanisms of TENG-based pulse sensors.

Structural Category	Representative Structures	Fabrication Methods	Structural Mechanism	Advantages
Interface micro/nanostructured	Nanowire arrays, dual-layer nanostructures, random micro-rough surfaces	Template replication, electrospinning, surface transfer	Amplifies weak arterial-pressure-induced deformation and effective contact area	High sensitivity at low pressure
Elastic support–regulated	In situ air gaps, sealed gas chambers, spacer-free contact–separation	In situ encapsulation, lamination	Enables large displacement and uniform stress distribution	Stable low-frequency response
Laminated thin-film	Multilayer flexible films	Casting/deposition + lamination	Global bending/compression-induced contact–separation	Simple structure, easy integration
Fiber/textile-configured	Fiber–fiber contact, core–sheath yarns, fabric–fabric stacking	Electrospinning, yarn spinning, weaving/knitting	Textile compressibility-induced multi-point contact–separation	Wearable, breathable, washable

## Data Availability

No new data were created or analyzed in this study. Data sharing is not applicable to this article.

## Abbreviations

The following abbreviations are used in this manuscript:

T-TENG	Textile-based Triboelectric Nanogenerator
TENG	Triboelectric Nanogenerator
F-TENG	Fiber-based Triboelectric Nanogenerator
Y-TENG	Yarn-based Triboelectric Nanogenerator
